# Differential Effects of Valence and Encoding Strategy on Internal Source Memory and Judgments of Source: Exploring the Production and the Self-Reference Effect

**DOI:** 10.3389/fpsyg.2019.01326

**Published:** 2019-06-12

**Authors:** Diana R. Pereira, Adriana Sampaio, Ana P. Pinheiro

**Affiliations:** ^1^Psychological Neuroscience Lab, CIPsi, School of Psychology, University of Minho, Braga, Portugal; ^2^Voice, Affect, and Speech Neuroscience Lab, Faculdade de Psicologia, Universidade de Lisboa, Lisbon, Portugal

**Keywords:** internal source memory, valence, emotion, production effect, self-reference effect, judgments of source, metamemory

## Abstract

Item memory studies show that emotional stimuli are associated with improved memory performance compared to neutral ones. However, emotion-related effects on source memory are less consistent. The current study probed how emotional valence and specific encoding conditions influence internal source memory performance and judgments of source (JOSs). In two independent experiments, participants were required to read silently/aloud (Experiment 1) or to perform self-reference/common judgments (Experiment 2) on a list of negative/neutral/positive words. They also performed immediate JOSs ratings for each word. The study phase was followed by a test phase in which participants performed old-new judgments. In Experiment 1, the production effect was replicated for item memory, but the effects of valence on item and source memory were not significant. In Experiment 2, self-referential processing effects on item and source memory differed as a function of valence. In both experiments, JOSs ratings were sensitive to valence and encoding conditions, although they were not predictive of objective memory performance. These findings demonstrate that the effects of valence on internal source memory and JOSs are modulated by encoding strategy. Thus, the way information is encoded can shed light on how emotion might enhance, impair or exert no influence on source memory.

## Introduction

Evidence for the intricate interactions between emotion and cognition has led to a remarkable shift in the cognitive sciences (Ochsner and Phelps, 2007; Okon-Singer et al., 2015). One example is the relationship between emotion and episodic memory. Specifically, the emotion-enhanced memory (EEM) effect refers to improved memory for emotionally charged (high arousal and/or negative/positive) compared to non-emotional (low arousal and/or neutral) information. This effect has been well documented for different stimulus types, such as single words (e.g., Kensinger and Corkin, 2003; D'Argembeau and Van der Linden, 2004; Davidson et al., 2006; Maddock and Frein, 2009), word pairs (e.g., Maddox et al., 2012), or pictures (e.g., Nashiro and Mather, 2011; Schmidt et al., 2011; Yick et al., 2015; Schümann et al., 2018). The EEM has also been extensively reported in the case of item memory (i.e., memory for the central features and relevant content of an event such as the words in a word pair; e.g., Kensinger and Schacter, 2008; Murphy and Isaacowitz, 2008; Levine and Edelstein, 2009; Kensinger and Kark, 2018). Nonetheless, a similar enhancement effect has been less consistent on other aspects of episodic memory such as source memory, i.e., the memory for the origins and conditions in which a certain event occurred (Johnson and Raye, 1981; Johnson et al., 1993; see Appendix for a selective review of these studies).

According to the source-monitoring framework (Johnson et al., 1993), when we try to specify the origins of an event, our decision may rely on qualitative characteristics that are bound to an item during encoding, including semantic detail, cognitive operations (e.g., elaboration; organization; imagery), perceptual (e.g., color; sound), contextual (e.g., time; space) and/or affective (e.g., emotional reactions) features, as well as on previous general knowledge or schemas (e.g., stereotypes; beliefs). The enhanced memory effect for items with emotional features during encoding has been discussed in light of different theoretical approaches, including the Easterbrook's (1959) cue-utilization hypothesis, the priority-binding theory (Mackay et al., 2004), the object-based binding theory (Mather, 2007), and the arousal-biased competition theory (Mather and Sutherland, 2011; see Bowen et al., 2018 for a review of other approaches). For instance, whereas impaired source memory for emotional stimuli might be explained by narrowed attention to central details and reduced capacity to bind central information with peripheral details (Easterbrook, 1959), a source memory enhancement might stem from a stronger activation of item-context binding mechanisms elicited by emotional events (Mackay et al., 2004). In turn, the object-based binding theory (Mather, 2007) conciliates contradictory reports of impairment vs. enhancement vs. null effects, by postulating that arousal facilitates the binding of elements that are an integral part of the emotional object (i.e., intrinsic features such as the color of the object), whereas it impairs or exerts no influence on the binding of an emotional object with other contextual information (i.e., extrinsic features such as the color of the border surrounding the object). Similarly, the arousal-biased competition theory (Mather and Sutherland, 2011) also predicts different outcomes for contextual details depending on the bottom-up (stimulus-driven information, such as its perceptual saliency) and/or top-down (goal-driven information, such as relevance for the current goals, expectations, and prior knowledge) priority of the information. In the latter case, arousal enhances memory for high priority information (i.e., information characterized by salient perceptual features and/or with relevance) while impairing memory for low priority information.

Even considering the former theoretical frameworks, several other factors may impact upon the relationship between emotion and source memory, including stimulus type (e.g., words or pictures; Phelps et al., 1997), the emotional properties of the stimuli (Boywitt, 2015), and the characteristics of the experimental task (e.g., intentional/incidental encoding, retention interval, encoding duration, type of memory test, sensory modality; D'Argembeau and Van der Linden, 2004; Ferré et al., 2019) . Additionally, other factors have been relatively ignored in the study of source memory and its interactions with emotion, such as the source monitoring task and the encoding strategy. Regarding the first, Johnson et al. (1993) proposed three distinct source monitoring processes: external source monitoring, which refers to the discrimination between different external sources (e.g., *something said speaker X or speaker Y?*); internal source monitoring, which relies on the distinction between internally derived information (e.g., *something I said silently or aloud?*); reality monitoring, which comprises the discrimination between internal and external sources (e.g., *something I heard/saw or imagined?*). Externally derived memories usually contain more perceptual, temporal, spatial, and affective information, whereas internally derived memories comprise more information regarding cognitive processes that occur during memory acquisition (Raye and Johnson, 1980; Johnson and Raye, 1981). Moreover, due to the overlap of informative cues in both quantity and quality, the discrimination between two or more external/internal sources is more challenging than the discrimination between an external and an internal source (Raye and Johnson, 1980). Accordingly, the way emotion affects source memory may depend on the contextual features that are manipulated, as already pointed out by previous studies showing that different external source memory tasks can lead to differences in source memory performance (e.g., D'Argembeau and Van der Linden, 2004; Koenig and Mecklinger, 2008; MacKenzie et al., 2015; Kuhlmann and Touron, 2017). For example, Boywitt (2015) demonstrated that the relationship between arousal and source memory performance is linear in the case of frame color, but it follows an inverted U-shape function for spatial location. Nonetheless, in comparison with external source memory, the study of other contextual features such as the discrimination between cognitive operations (internal source memory) when using emotional stimuli has received less attention (see Appendix).

In the case of encoding strategies, different approaches have been shown to benefit source memory performance such as semantic clustering (Wegesin et al., 2000) and unitization (i.e., the ability to bind items or item-context in a single and meaningful combination; Tu and Diana, 2016; Tu et al., 2017; see also El Haj et al., 2016 for a review). Additionally, Kuhlmann and Touron (2012, 2017) observed that when participants spontaneously used interactive imagery and sentence generation during encoding, source memory performance was improved. Notwithstanding, other encoding factors already known to boost item memory, such as production mode and self-referential processing, may also influence source memory (Hamami et al., 2011; Serbun et al., 2011; Leshikar and Duarte, 2012, 2014; Ozubko et al., 2014; Yin et al., 2018). Specifically, the production effect is a simple mnemonic strategy that shows a memory benefit for vocal production conditions, such as mouthing, reading aloud, reading aloud loudly, and singing, when compared to silent reading conditions (Dodson and Schacter, 2001; MacLeod et al., 2010; Ozubko and MacLeod, 2010; Quinlan and Taylor, 2013). The self-reference effect, in turn, represents a memory benefit for information that is encoded in relation to the self in comparison with information processed in other deep (e.g., semantic; other self-referent) or shallow (e.g., phonemic; perceptual) conditions (Rogers et al., 1977; Kuiper and Rogers, 1979; Symons and Johnson, 1997; Leshikar and Duarte, 2012; Yang et al., 2012; Leshikar et al., 2015).

Acknowledging the modulatory role of the encoding strategy and type of source memory task in the interaction between emotion and source memory, the first aim of the current study was to explore how stimulus valence influences internal source monitoring, when the encoding strategies (production mode and the self-referential processing) are also manipulated. Regarding the production mode, previous studies revealed that a similar beneficial effect is observed on item and source memories when stimuli are produced aloud (Ozubko et al., 2014). Nonetheless, to the best of our knowledge, no previous study has concomitantly explored the role of emotion and production mode in source memory. In the case of the self-reference effect, previous research demonstrated that both item and source memory are enhanced when a self-referential approach is implemented during encoding (Hamami et al., 2011; Serbun et al., 2011; Leshikar and Duarte, 2012, 2014; Kalenzaga et al., 2015; Yin et al., 2018; Zhang et al., 2018). Also, when stimulus valence is manipulated, both neutral and positive stimuli that are self-referentially encoded are better remembered compared to negative stimuli (e.g., D'Argembeau et al., 2005; Yang et al., 2012; Leshikar et al., 2015; Zhang et al., 2018). This pattern appears to hold partially for positive stimuli in the case of internal source monitoring, but it has been less consistently explored in the case of neutral stimuli (e.g., Durbin et al., 2017). Thus, in the current study, we expected to replicate the self-reference benefit for both item and source memory, and to better qualify this effect when valence is manipulated during encoding.

Together with objective measures of memory performance, a second aim of this study was to examine how stimulus valence and encoding strategy might modulate subjective metamemory processes, specifically judgements of source (JOSs). Metamemory encompasses specific knowledge and beliefs about memory functioning (Flavell, 1979), such as what we will remember or forget in the future. The study of metamemory often relies on prospective tasks in which participants are required to judge if they will remember/forget previously learned information, or on retrospective tasks in which participants are asked to evaluate their memory performance after remembering some information (Nelson and Narens, 1990). One of the most common measures to assess prospective metamemory relies on judgments of learning (JOLs), in which participants are required to make a prediction about the memorability of specific stimuli, during or after their acquisition, through a rating or numeric scale. So far, few studies have explored the role of emotion on metamemory judgments. The existing evidence suggests that participants deem emotional stimuli to be more memorable than neutral stimuli during the encoding stage. This effect has been observed with odors (Jönsson et al., 2005), faces (Nomi et al., 2013), pictures (Hourihan and Bursey, 2017), and words (Zimmerman and Kelley, 2010; Tauber and Dunlosky, 2012; Hourihan et al., 2017). When considering the influence of emotion on judgments of source (JOSs), the existing data is even more scarce. One of the first studies to introduce the concept of JOSs was led by Dutton and Carroll (2001), in which they explored how three different emotionally arousing conditions modulated both JOLs and JOSs predictions. These authors found that JOLs and JOSs predictions somehow matched the recall performance, particularly in emotional conditions characterized by high arousal. In this context, the results of the current study can represent a novel contribution to the study of emotion and prospective metamemory, especially in the case of internal source memory monitoring.

Based on the evidence reviewed above, our first experiment investigated possible interactions between the production effect and stimulus valence in the case of internal source monitoring and JOSs. First, we predicted a replication of the EEM for item memory (Kensinger and Schacter, 2008; Murphy and Isaacowitz, 2008; Levine and Edelstein, 2009; Kensinger and Kark, 2018), i.e., better recognition performance for both negative and positive stimuli in comparison with neutral stimuli regardless of the production mode, based on the assumption that both production modes recruit similar attentional and elaborative processes. Second, in the case of internal source memory, we hypothesized an impairment or, more likely, a null effect of valence (see Appendix for a selective review of studies). This prediction is supported by the object-based binding hypothesis (Mather, 2007) as the production mode can be deemed as an extrinsic feature of the stimuli. It is also supported by the arousal-biased competition theory (Mather and Sutherland, 2011): considering that participants were overtly instructed to memorize the stimuli in the current experiment, both emotional and non-emotional stimuli were goal-relevant and received similar top-down priority. Third, we expected JOSs to be sensitive to both mode of production and stimulus valence as previous studies demonstrated that stimuli read aloud receive higher JOLs ratings than stimuli read silently (Castel et al., 2013), and that emotional words are perceived as more memorable (Zimmerman and Kelley, 2010; Tauber and Dunlosky, 2012; Hourihan et al., 2017). In our second experiment, the effects of both self-referential encoding and valence on internal source memory and JOSs were examined. The replication of the self-reference mnemonic benefit for both item and source memory was expected, especially in the case of neutral and positive stimuli (D'Argembeau et al., 2005; Yang et al., 2012; Leshikar et al., 2015; Zhang et al., 2018). Although these predictions contrast with reports of null or impairment effects of emotion on internal source memory (see Appendix), they highlight how the encoding strategy and top-down processes, such as motivational relevance (Mather and Sutherland, 2011), might modulate the relationship between emotion and source memory. Regarding the metamemory judgments, we hypothesized JOSs to be sensitive to valence and encoding strategy as in Experiment 1. Specifically, we predicted that positive and neutral stimuli would be judged as more memorable than negative stimuli given that they might be perceived as more relevant self-descriptors (Kuiper and Rogers, 1979; D'Argembeau et al., 2005).

## Experiment 1

In a previous study by Ozubko et al.(2014, Experiment 3), the production mode benefited internal source memory for items read aloud during encoding. Reading aloud seems to be a distinctive feature that can function as a diagnostic cue during the test phase: if we remember that a specific word was read aloud, we will probably classify the item as “old”. This will be less likely to occur in the case of silent words as they have the same production status as new words during the test (MacLeod et al., 2010; Ozubko and MacLeod, 2010). In the current experiment, the source memory benefit for items read aloud was tested by manipulating both the production mode (silent vs. aloud) and stimulus valence (negative vs. neutral vs. positive). During the study phase, participants were required to read a list of words with different valence properties, half aloud and half silently. For each word, participants were also asked to make JOSs using a six-point rating scale. Immediately after learning, participants performed a test in which they were asked to discriminate between old and new items and to recall the production mode.

### Material and Methods

#### Participants

Thirty-two college students participated in this experiment. However, one was excluded due to self-reported depression diagnosis and use of antidepressants; three other participants were excluded as they scored above 13 in the Portuguese version of the Beck Depression Inventory-II, which is indicative of depressive symptoms (Coelho et al., 2002). The final sample was composed of 28 participants (23 females), aged between 18 and 32 years (*M* = 20.25, *SD* = 3.62), and with an average of 13.54 years of formal education (*SD* = 2.28). There were no self-reports of psychiatric/neurological disorders nor psychoactive drug usage. Participants had normal or corrected-to-normal vision, and they reported no auditory or other sensory and motor problems. They provided informed consent prior to their enrolment in the study and received course credit for their collaboration. The research protocol was approved by the local Ethics Committee (University of Minho, Braga, Portugal).

In Experiment 3 of Ozubko et al. (2014), where a superiority source memory effect was found for items studied aloud, a total of 24 participants were enrolled and the effect sizes (partial eta squared - $\eta_{\text{p}}^{2}$) ranged between 0.24 and 0.77. Based on this information, *a priori* sample size estimation was conducted using G^*^Power-3 statistical software (Faul et al., 2007), considering the within-factors repeated-measures analysis of variance (RMANOVA; 3 valence: negative/neutral/positive x 2 source: aloud/silent design) as the main statistical test. For an alpha significance level of 0.05, a power of 0.80, and an effect size of 0.24 (as calculated in SPSS), a sample of at least 11 participants would be required to allow the detection of differences between modes of production in source memory. Thus, the number of recruited participants was adequate.

#### Materials

A total of 180 words (60 negative/neutral/positive; see Table S1) were initially selected from the Portuguese version of the Affective Norms for English Words (ANEW; Soares et al., 2012). Words differed in valence, ranging from 1 to 9, with 9 corresponding to the most positive ratings and 1 to the least positive ratings (positive > neutral; positive > negative; neutral > negative; all *p* < 0.001). In the case of arousal, differences were observed between neutral and both negative and positive stimuli (neutral < negative; neutral < positive; negative = positive; all *p* < 0.001). No significant differences were found in frequency, number of letters and number of syllables as a function of word valence (*p* > 0.05; see Table S1). The selected words were randomly distributed across six lists of 30 stimuli each (with 10 stimuli of each valence category) to be used as learning and test items. Lists did not differ in words' valence, arousal, frequency, number of letters, and number of syllables (*p* > 0.05).

#### Procedure

**Figure 1:**
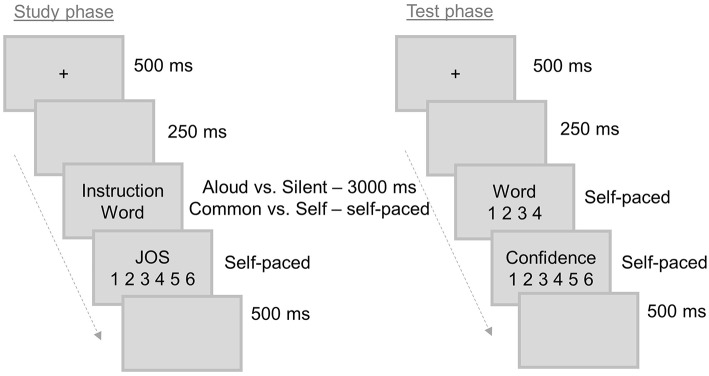
Schematic illustration of the tasks used in Experiments 1 and 2. Note: JOS, Judgement of Source.

The experimental task was completed in a single session, and it consisted of three study-test cycles. During the study phase, each trial began with a fixation cross (500 ms) followed by a blank screen (250 ms), and then a target word (3,000 ms), which was presented in the center of the screen in a light gray background (Arial, black color, font size 28). Together with each word, the instruction “read silently” or “read aloud” was presented on top of the screen to inform how the word should be read. Participants were then instructed to judge how likely they were to remember that the previous word was read silently or aloud by using a six-point rating scale following Hourihan et al. (2017), in which “1” represented “Sure I will not remember” and “6” represented “Sure I will remember.” The rating scale remained on the screen until the participant made the JOSs. Lastly, a blank screen appeared during 500 ms (see Figure 1). Thirty stimuli were randomly presented during encoding to be later tested. Four additional words, two at the beginning and two at the end of the list, were used as fillers to mitigate possible primacy and recency effects, but they were not considered in the analysis. No specifications were provided about which fingers the participants should use to provide a response.

The study phase was immediately followed by a test phase in which the previously 30 read words were randomly mixed with 30 new words. A trial started with a fixation cross (500 ms) and a blank screen (250 ms); then a word was presented in the center of the screen, and participants had to perform old-new judgments regarding the source by choosing one of four response options: (1) “read silently”; (2) “read aloud”; (3) “read, but do not know if silently/aloud”; (4) “new”. The “do not know” option was adopted following previous studies (Sharot and Yonelinas, 2008; Leshikar and Duarte, 2012, 2014; Newsome et al., 2012; Dulas and Duarte, 2014) with the purpose of reducing guessing and response bias. There was no time limit to provide a response. The response keys were counterbalanced across participants. After the old-new judgment, participants were asked to evaluate the degree of confidence in their response using, once again, a six-point rating scale (1 = “No confidence”; 6 = “Complete confidence”). The confidence judgment was also self-paced. The last event of the trial was a blank screen lasting 500 ms (see Figure 1).

The lists were counterbalanced across participants regarding study/test and read aloud/silent to ensure that each stimulus could be read aloud or silently and could be an old or a new item. Moreover, prior to the experimental task, all participants completed a training period with a brief study-test cycle, and they were instructed to pay attention to the words and their source (aloud or silent), because they would be tested later (intentional learning). Participants were also informed that each study-test cycle was independent, i.e., after finishing a cycle they could forget the stimuli and focus their attention on the new cycle. Stimulus presentation was controlled using SuperLab software (Version 5, Cedrus Corporation, San Pedro, CA, https://www.cedrus.com/superlab/).

#### Data Analysis

Initially, the proportion of responses during recognition was calculated for six main categories: (1) correct source (the source was correctly identified; for example, the participant recognized an item as “read aloud” when it was indeed paired with a “read aloud” instruction during the study phase); (2) incorrect source (the participant misattributed the source; for example, for an item that was paired with a “read aloud” instruction during encoding, the participant selected “read silently” during test); (3) do not know source (the participant correctly recognized an item as old, but did not remember if it was read silently or aloud); (4) miss (for an item presented during the study phase, the participant misidentified it as “new” during test); (5) correct rejection (the participant correctly identified a new item as “new”); (6) false alarm (the participant considered a new item as “old” by choosing one of the sources or the “do not know” option). The item memory recognition accuracy was then obtained using Pr = ([p(hits) – p(false alarms)]) (Snodgrass and Corwin, 1988), wherein the p(hits) resulted from combining both correct and incorrect source responses and the do not know responses. The response bias also followed Snodgrass and Corwin (1988): Br = ([p(false alarms)/(1 – Pr)]). A conservative response bias is considered for Br values below 0.50, whereas a liberal response bias is considered for values above 0.50. In a first attempt to compute the Br, the denominator was equal to zero for some participants. To circumvent this problem, we applied a correction to both hit and false alarm rates, following the same authors [hit rates = (number of hits + 0.5) / (number of old items + 1); false alarm rates = (number of false alarms + 0.5) / (number of new items + 1)]. In the case of source recognition, the discrimination measure was obtained by subtracting incorrect source from correct source responses - [p(correct source) – p(incorrect source)] – excluding the “do not know” responses and following prior studies (e.g., Newsome et al., 2012; Dulas and Duarte, 2014; Leshikar et al., 2015). Both item and source discrimination measures were submitted to a 3 (valence: negative/neutral/positive) x 2 (source: aloud/silent) RMANOVA.

It is worth noting that the old-new recognition patterns and the source memory discrimination are somehow superimposed and confounded in this study. Moreover, the computed measures are amenable to response bias. Multinomial models represent a useful approach to separate between old-new detection and source discrimination, by additionally considering the effect of potential biases (Batchelder and Riefer, 1990). Thereby, multinomial models were computed as a supplementary analysis to back up the results of the RMANOVA. A full description of the procedure and results is presented in Supplementary Material.

The effects of source and emotional content on the JOSs during encoding were evaluated through a 3 (valence) x 2 (source) RMANOVA. Moreover, Goodman-Kruskal gamma correlations were used as indices of metamnenomic accuracy for each experimental condition in accordance with previous literature (e.g., Castel et al., 2013). The procedure used to compute gamma is described in Supplementary Material. Additional findings regarding the proportion of incorrect source responses, “do not know” responses, misses, correct rejections, (corrected) false alarms, recognition confidence for source judgments and correct rejections, as well as the response times for JOSs and recognition responses are presented in Supplementary Material.

A Bayesian two-way RMANOVA was conducted (JASP Team, 2018, Version 0.9.0.1) to complement the abovementioned analysis (Wagenmakers et al., 2018a,b see Tables S7, S8). Specifically, Bayes factors (BF_10_) were considered in favor of the alternative hypothesis if BF_10_ was larger than three, whereas a BF_10_ lower than 0.3 was interpreted as evidence in favor of the null hypothesis (Quintana and Williams, 2018; Wagenmakers et al., 2018a). Finally, the Greenhouse-Geisser method was used as correction for sphericity violations, and Bonferroni-corrected *post hoc* comparisons were applied to qualify interaction effects.

### Results

Descriptive statistics regarding the behavioral performance in Experiment 1 are shown in Table 1.

**Table 1:** Behavioral performance (source x valence) in Experiments 1 and 2.

	**Experiment 1–*****M*** **(*****SD*****)**						**Experiment 2–*****M*** **(*****SD*****)**					
	**Aloud**			**Silent**			**Self**			**Common**		
	**Negative**	**Neutral**	**Positive**	**Negative**	**Neutral**	**Positive**	**Negative**	**Neutral**	**Positive**	**Negative**	**Neutral**	**Positive**
Source correct	0.61 (0.16)	0.55 (0.20)	0.58 (0.19)	0.59 (0.22)	0.57 (0.24)	0.55 (0.24)	0.64 (0.19)	0.76 (0.20)	0.79 (0.17)	0.47 (0.23)	0.58 (0.26)	0.44 (0.24)
Source incorrect	0.14 (0.12)	0.11 (0.14)	0.11 (0.14)	0.05 (0.08)	0.05 (0.08)	0.05 (0.08)	0.08 (0.10)	0.05 (0.09)	0.06 (0.10)	0.08 (0.08)	0.04 (0.06)	0.14 (0.12)
Source DNK	0.18 (0.17)	0.22 (0.19)	0.21 (0.16)	0.24 (0.17)	0.21 (0.22)	0.22 (0.17)	0.14 (0.14)	0.08 (0.10)	0.08 (0.11)	0.19 (0.20)	0.17 (0.19)	0.21 (0.18)
Source miss	0.07 (0.10)	0.11 (0.08)	0.09 (0.08)	0.12 (0.12)	0.17 (0.16)	0.17 (0.14)	0.15 (0.13)	0.11 (0.10)	0.07 (0.11)	0.25 (0.15)	0.21 (0.15)	0.21 (0.17)
Item Pr measure	0.82 (0.13)	0.78 (0.13)	0.76 (0.13)	0.77 (0.15)	0.72 (0.16)	0.68 (0.15)	0.68 (0.14)	0.76 (0.14)	0.74 (0.17)	0.59 (0.15)	0.67 (0.17)	0.60 (0.20)
Item Br measure	0.47 (0.22)	0.36 (0.20)	0.48 (0.26)	0.38 (0.23)	0.33 (0.25)	0.39 (0.23)	0.43 (0.25)	0.43 (0.23)	0.57 (0.26)	0.33 (0.21)	0.33 (0.21)	0.39 (0.26)
Source measure	0.46 (0.20)	0.44 (0.27)	0.46 (0.27)	0.54 (0.24)	0.52 (0.26)	0.49 (0.27)	0.56 (0.25)	0.71 (0.26)	0.72 (0.22)	0.40 (0.27)	0.54 (0.28)	0.30 (0.31)
JOSs ratings	4.40 (0.68)	4.02 (0.71)	4.26 (0.76)	3.56 (0.63)	3.25 (0.52)	3.50 (0.60)	4.05 (0.85)	4.12 (0.85)	4.34 (0.82)	3.91 (0.72)	3.94 (0.69)	3.96 (0.79)
Gamma (JOSs)	0.18 (0.53)	0.29 (0.51)	0.31 (0.49)	0.19 (0.45)	0.19 (0.58)	0.14 (0.48)	0.15 (0.47)	0.16 (0.50)	0.14 (0.52)	0.11 (0.53)	0.06 (0.45)	0.07 (0.48)
	Negative		Neutral		Positive		Negative		Neutral		Positive	
CR	0.92 (0.09)		0.93 (0.10)		0.89 (0.11)		0.88 (0.12)		0.91 (0.09)		0.85 (0.15)	
FA	0.08 (0.09)		0.07 (0.10)		0.11 (0.11)		0.12 (0.11)		0.09 (0.09)		0.15 (0.15)	

*Note: CR, correct rejections; DNK, do not know; FA, false alarms; JOSs, judgments of source*.

#### Recognition Accuracy

##### Item recognition

**Figure 2:**
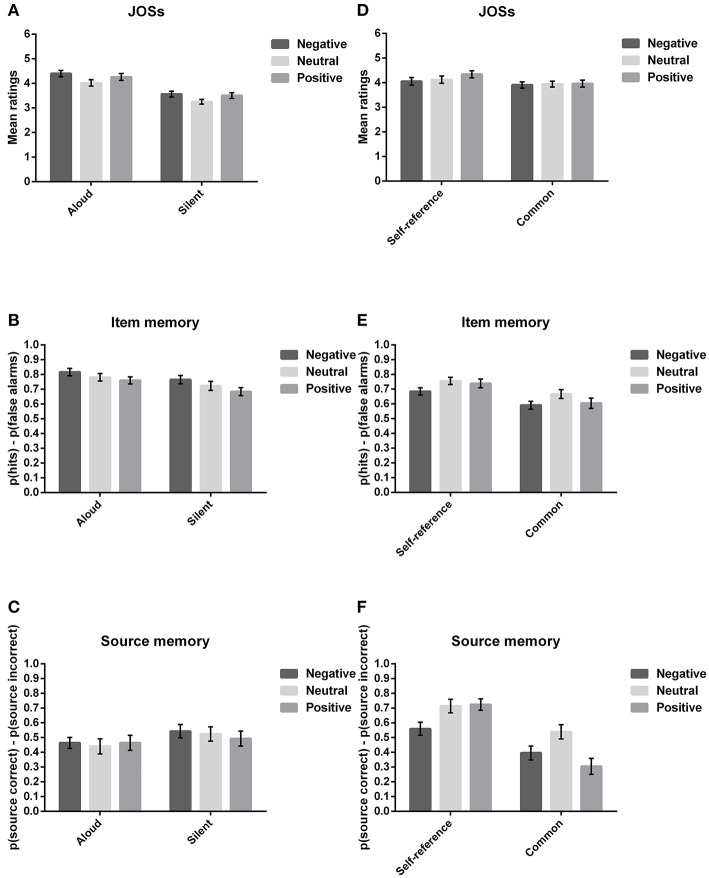
Behavioral results for JOSs, item memory and source memory in Experiments 1 and 2. Specifically, the behavioral results for judgments of source (JOSs**; A**,**D**), item memory **(B,E)**, and source memory **(C,F)** are plotted on the y-axis as a function of source (aloud/silence; self-reference/common) and valence (negative/neutral/positive). Note: The error bars indicate the standard errors of the means.

The RMANOVA yielded a main effect of valence, *F*_(2, 54)_ = 4.08, *p* = 0.022, $\eta_{\text{p}}^{2}$ = 0.13, a main effect of source, *F*_(1, 27)_ = 15.93, *p* < 0.001, $\eta_{\text{p}}^{2}$ = 0.37, but no interaction effect between the two factors, *F*_(2, 54)_ = 0.34, *p* = 0.716, $\eta_{\text{p}}^{2}$ = 0.01. Bayes factors (see Table S7) favored the two main effects model in relation to the null model (BF_10_ = 671.057 ± 2.21%). The comparison between this model and the model that adds the interaction term (671.247/82.160 = 8.17) revealed evidence against the interaction as data were 8.17 more likely in the two main effects model than in the model with the interaction. Specifically, the pairwise comparisons showed a marginally significant difference in the recognition of negative and positive words (*p* = 0.060, *d* = 0.47, 95% CI [-0.002, 0.14]): negative words (*M* = 0.79, *SE* = 0.03) were more accurately recognized than positive words (*M* = 0.72, *SE* = 0.02). The words read aloud (*M* = 0.79, *SE* = 0.02) were also more accurately recognized than words read silently (*M* = 0.72, *SE* = 0.02; *p* < 0.001, *d* = 0.75, 95% CI [0.03, 0.10]; see Table 1 and Figure 2B).

In the case of the corrected item Br measure, the RMANOVA showed a main effect of valence *F*_(2, 54)_ = 3.64, *p* = 0.033, $\eta_{\text{p}}^{2}$ = 0.12, a main effect of source, *F*_(1, 27)_ = 11.79, *p* = 0.002, $\eta_{\text{p}}^{2}$ = 0.30, yet no interaction effect, *F*_(2, 54)_ = 1.03, *p* = 0.363, $\eta_{\text{p}}^{2}$ = 0.04. Again, the Bayesian analysis supported the two main effects model when compared to the null model (BF_10_ = 42.206 ± 1.50%). Additionally, when compared to the model with the interaction term (42.206/7.965), the data were 5.30 times more likely in the two main effects model (see Table S7). Specifically, there was a statistically significant difference between neutral and positive words (*p* = 0.035, *d* = 0.51, 95% CI [0.005, 0.16]), indicating that participants used a less conservative response criterion for positive words (*M* = 0.43, *SE* = 0.04) in comparison with neutral words (*M* = 0.35, *SE* = 0.04). Also, the response criterion was less conservative for words read aloud (*M* = 0.44, *SE* = 0.04) than for words read silently during encoding (*M* = 0.37, *SE* = 0.04; *p* = 0.002, *d* = 0.65, 95% CI [0.029, 0.12]). However, the response criterion for all conditions was on average below or near 0.50, thus indicating a general conservative to neutral response bias (see Table 1; Snodgrass and Corwin, 1988).

##### Source recognition

The results regarding the source memory measure did not follow the same pattern as the item memory, as no statistically significant effects were observed [valence: *F*_(2, 54)_ = 0.42, *p* = 0.657, $\eta_{\text{p}}^{2}$ = 0.02; source: *F*_(1, 27)_ = 1.37, *p* = 0.252, $\eta_{\text{p}}^{2}$ = 0.05; interaction: *F*_(2, 54)_ = 0.49, *p* = 0.618, $\eta_{\text{p}}^{2}$ = 0.02)]. Bayesian analysis supported the null effects (valence: BF_10_ = 0.072 ± 0.86%; source: BF_10_ = 1.027 ± 4.28%; valence + source + interaction: BF_10_ = 0.009 ± 1.75%). Thus, the source memory recognition measures were similar irrespective of stimulus valence and of the encoding strategy (see Table 1 and Figure 2C).

#### Judgments of Source (JOSs)

When assessing how valence and way of production affected the JOSs ratings, we observed a main effect of valence, *F*_(2, 54)_ = 15.93, *p* < 0.001, $\eta_{\text{p}}^{2}$ = 0.37, a main effect of source, *F*_(1, 27)_ = 59.61, *p* < 0.001, $\eta_{\text{p}}^{2}$ = 0.69, but no interaction effect, *F*_(2, 54)_ = 0.44, *p* = 0.649, $\eta_{\text{p}}^{2}$ = 0.02. The Bayes factors supported the two main effects model with no interaction (valence + source: BF_10_ = 1.620 x 10^23^ ± 1.25%; 8.29 times more likely than the interaction model). Specifically, the words read aloud (*M* = 4.23, *SE* = 0.13) were regarded as more memorable than the words read silently (*M* = 3.44, *SE* = 0.10; *p* < 0.001, *d* = 1.46, 95% CI [0.58, 1.00]), and both negative (*M* = 3.98, *SE* = 0.11; *p* < 0.001, *d* = 1.05, 95% CI [0.19, 0.50]) and positive stimuli (*M* = 3.88, *SE* = 0.12; *p* = 0.001, *d* = 0.80, 95% CI [0.10, 0.40]) were rated as more memorable when compared to neutral stimuli (*M* = 3.64, *SE* = 0.10; see Table 1 and Figure 2A).

In general, the metacognitive judgments partially agree with the results of item memory, as words read aloud were better recognized than words read silently. However, the predictions in the case of valence were less accurate, since no significant differences were found between emotional conditions. Considering that the JOSs ratings were performed in relation to the source, no match was observed between the predictions and the source memory performance. The analysis of the metamnemonic accuracy revealed that the gamma correlations for neutral/positive words that were read aloud and for negative words read silently were statistically different from zero, *t*_(27)_ ≥ 2.29, *p* ≤ 0.03, whereas for the remaining conditions the gamma correlations did not differ from zero, *t*_(27)_ ≤ 1.78, *p* ≥ 0.09. On the one hand, some experimental conditions were different from zero, supporting a possible relation between immediate metamemory judgments and recognition. Additionally, even though the mean gamma coefficients were positive and small (see Table 1), they resemble what has been reported in the literature of metamnemonic accuracy in the case of source memory (e.g., Carroll et al., 1999). On the other hand, no statistically significant differences between conditions were observed for gamma correlations (valence: *F*_(2, 54)_ = 0.14, *p* = 0.866, $\eta_{\text{p}}^{2}$ = 0.01; source: *F*_(1, 27)_ = 1.06, *p* = 0.313, $\eta_{\text{p}}^{2}$ = 0.04; interaction: *F*_(2, 54)_ = 0.61, *p* = 0.549, $\eta_{\text{p}}^{2}$ = 0.02). Bayes factors were also in agreement (valence: BF_10_ = 0.069 ± 0.86%; source: BF_10_ = 0.296 ± 1.18%; valence + source + interaction: BF_10_ = 0.003 ± 2.86%). Thus, the modulatory role of encoding strategy and valence in the associations between the prospective source predictions and the actual source memory performance was inconclusive.

### Discussion: Experiment 1

A major finding of Experiment 1 was the absence of a production effect on internal source memory. Nonetheless, as expected, the effect was replicated in the case of item memory (Dodson and Schacter, 2001; MacLeod et al., 2010; Ozubko and MacLeod, 2010; Quinlan and Taylor, 2013). Moreover, there was no interaction between valence and production mode, indicating that the production effect on item memory occurred regardless of whether stimuli had or not an emotional quality. The EEM on item memory was also absent, against our predictions and the main tendency in the literature (see Kensinger and Schacter, 2008; Murphy and Isaacowitz, 2008; Levine and Edelstein, 2009, and Kensinger and Kark, 2018, for overviews), especially if we take into consideration that both positive/negative words were rated as significantly more arousing than neutral words. Even so, this result is not unprecedented (e.g., Hourihan and Bursey, 2017; Ferré et al., 2019). Considering the response bias, participants revealed a conservative to neutral response criterion (see Table 1). Specifically, in cases of uncertainty, participants showed an increased tendency to regard an item as “new” when it was studied in the “read silently” condition than when it was studied in the “read aloud” condition. A similar tendency was observed for neutral items when compared to positive items. Although valence did not significantly affect objective memory indices, the same was not observed for subjective metamemory judgements. In fact, participants regarded the source of emotional words that were read aloud as more memorable, providing support to previous studies with JOLs (Jönsson et al., 2005; Zimmerman and Kelley, 2010; Tauber and Dunlosky, 2012; Castel et al., 2013; Nomi et al., 2013; Hourihan et al., 2017). However, contrary to the findings of Dutton and Carroll (2001), the JOSs predictions were not associated with internal source memory performance, which favors the notion that metamemory judgments are not always predictive of future memory (e.g., Carroll et al., 1999). Rather, they show that participants were sensitive to variations in the encoding conditions, and that they used these cues to perform metamemory judgments (Mazzoni and Nelson, 1995; Koriat, 1997; Nomi et al., 2013).

## Experiment 2

The experimental design was identical to Experiment 1, although a different encoding strategy was tested: the self-referential processing. Given that in Experiment 1 negative and positive words were more arousing than neutral words, in Experiment 2 arousal ratings were also equated across valence categories. The control of arousal is advantageous considering that the effects of valence and arousal on memory function appear to be supported by different processing mechanisms (valence effects have been associated with more effortful semantic and autobiographical elaboration processes, whereas arousal effects have been related to more automatic processes; Kensinger and Corkin, 2004; Cook et al., 2007). Moreover, these differential effects also seem to be supported by distinct neurofunctional mechanisms (prefrontal cortex-hippocampal interactions in the case of valence, and amygdala-hippocampal interactions in the case of arousal; Kensinger and Corkin, 2003, 2004). Hence, the control of arousal allows isolating valence-related effects. Specifically, in this experiment, emotional (negative/positive) and neutral adjectives with medium arousal ratings were used as stimuli. During the encoding phase, participants performed two types of judgments. In half the trials, they were required to judge if the word was related to them. In the other half, they were asked if the word was commonly used by people in everyday life. The latter task represents a non-self-referential condition that requires a deep, semantic analysis of the word, and it was based on previous research (e.g., Hamami et al., 2011; Serbun et al., 2011; Newsome et al., 2012; Leshikar et al., 2015). Participants were also instructed to perform JOSs for each word. During the test phase, participants performed old-new judgments in which they were additionally asked to identify the source of the item (self-reference vs. common).

### Materials and Methods

#### Participants

A total of 32 young adults (30 females), aged between 18 and 45 years (*M* = 22.59, *SD* = 5.89), and with an average of 14.45 years of formal education (*SD* = 2.35) participated in this experiment. The same inclusion criteria described in Experiment 1 were applied to Experiment 2. The sample size estimation followed the same strategy as described in Experiment 1 (i.e., within factors RMANOVA as the main statistical test, $\eta_{\text{p}}^{2}$ as measure of effect size, similar experimental design, and the same parameters of alpha significance level and power), but a recent study by Durbin et al. (2017, Experiment 1) was used as reference: here, an effect size of 0.16 was reported for source memory performance (24 participants were tested). G^*^Power (Faul et al., 2007) indicated that a minimum of 16 participants would be necessary to allow the detection of such effects. Hence, an adequate sample size was also tested in the current experiment.

#### Materials

A total of 144 adjectives (48 negative/neutral/positive) were selected from the Portuguese version of the ANEW (Soares et al., 2012). The valence ratings differed between the three types of words (positive > neutral; positive > negative; neutral > negative; all *p* < 0.001), but no further differences were observed regarding arousal, frequency, number of letters and number of syllables (*p* > 0.05; see Table S1). The selected stimuli were then equally divided among four lists of 36 words each (12 stimuli from each valence category) to be used in the study and test phases. The lists were similar regarding valence, arousal, frequency, number of letters, and number of syllables (*p* > 0.05).

#### Procedure

The overall procedure, structure, and characteristics of the experimental task were similar to Experiment 1 (see Figure 1), but with the following differences: (a) only two study-test cycles were used; (b) during the encoding phase, the participants performed two different subjective judgments—a self-referential judgment (in which they assessed whether each stimulus related somehow to their personal characteristics: self-referential condition) or a common judgment (in which they evaluated whether the word was commonly used by people in their everyday lives: non-self-referential condition); (c) the instructions that appeared together with the words were “Does this word describe me?” or “Is this word common?”; d) during the encoding phase, the participants were prompted to respond “yes” (press key “Z”) or “no” (press key “M”) in accordance to the “self-reference” or “common” instructions, and there was no time limit to produce a response (self-paced); e) during the test phase, the participants were instructed to select one of four response options: “self-description”; “common”; “evaluated, but do not know if self-description/common”; “new”.

#### Data Analysis

The same data analysis procedures planned for Experiment 1 were adopted in Experiment 2.

### Results

The main descriptive statistics for the behavioral performance in Experiment 2 are shown in Table 1.

#### Recognition Accuracy

##### Item memory

The analysis of the corrected item memory Pr measure showed a main effect of valence, *F*_(2, 62)_ = 5.46, *p* = 0.012, $\eta_{\text{p}}^{2}$ = 0.15, ε = 0.78, a main effect of source, *F*_(1, 31)_ = 41.63, *p* < 0.001, $\eta_{\text{p}}^{2}$ = 0.57, but no valence x source interaction effect, *F*_(2, 62)_ = 1.15, *p* = 0.314, $\eta_{\text{p}}^{2}$ = 0.04, ε = 0.76. The two main factors model received the strongest support (BF_10_ = 8.708 × 10^7^ ± 1.18%; see Table S8), and it was preferred to the interaction model (8.708 x 10^7^/1.582 x 10^7^ = 5.50). The participants were better at recognizing neutral words (*M* = 0.71, *SE* = 0.03) relative to negative words (*M* = 0.64, *SE* = 0.02; *p* < 0.001, *d* = 0.82, 95% CI [0.03, 0.11]). They also demonstrated a better recognition performance for words encoded in the self-referential condition (*M* = 0.73, *SE* = 0.02) than for words studied in the common condition (*M* = 0.62, *SE* = 0.03; *p* < 0.001, *d* = 1.14, 95% CI [0.07, 0.14]; see Table 1 and Figure 2E).

Regarding the corrected item Br measure, the RMANOVA revealed a main effect of valence, *F*_(2, 62)_ = 9.26, *p* < 0.001, $\eta_{\text{p}}^{2}$ = 0.23, a main effect of source, *F*_(1, 31)_ = 40.97, *p* < 0.001, $\eta_{\text{p}}^{2}$ = 0.57, yet no interaction effect, *F*_(2, 62)_ = 2.33, *p* = 0.106, $\eta_{\text{p}}^{2}$ = 0.07. Again, Bayes Factors were in favor of the two main effects model in contrast to the null model (BF_10_ = 3.092 x 10^8^ ± 2.06%; see Table S8). This model was also favored when compared to the model with the interaction term (3.092 x 10^8^/1.201 x 10^8^ = 2.57). Specifically, the participants demonstrated a more conservative response criterion for both negative (*M* = 0.38, *SE* = 0.04) and neutral words (*M* = 0.38, *SE* = 0.02) when compared to positive words (*M* = 0.48, *SE* = 0.04; *p* = 0.003, *d* = 0.63/0.64, 95% CI [0.03, 0.18] considering both comparisons). Moreover, participants used a less conservative response criterion for words encoded in the self-referential condition (*M* = 0.48, *SE* = 0.04) compared to words studied in the common condition (*M* = 0.35, *SE* = 0.04; *p* < 0.001, *d* = 1.13, 95% CI [0.09, 0.17]; see Table 1).

##### Source memory

The RMANOVA yielded a main effect of valence, *F*_(2, 62)_ = 15.17, *p* < 0.001, $\eta_{\text{p}}^{2}$ = 0.33, ε = 0.79, a main effect of source, *F*_(1, 31)_ = 39.58, *p* < 0.001, $\eta_{\text{p}}^{2}$ = 0.56, and a valence x source interaction effect, *F*_(2, 62)_ = 10.69, *p* < 0.001, $\eta_{\text{p}}^{2}$ = 0.26. Positive evidence in favor of the interaction model over the two main effects model was also obtained with Bayes factors (1.007 × 10^13^/9.218 × 10^14^ = 0.01). Specifically, the interaction effect yielded a differential pattern of results when considering the self-referential and the common condition. Whereas, the source of negative words encoded in the self-referential condition was more poorly recognized than the source of both neutral (95% CI [0.07, 0.23]) and positive words (95% CI [0.05, 0.28]), the source of both negative (95% CI [0.04, 0.25]) and positive words (95% CI [0.13, 0.34]) was less accurately recognized than the source of neutral words in the case of the common condition (*p* < 0.01; see Table 1 and Figure 2F).

#### Judgments of Source (JOSs)

Concerning the JOSs ratings, the RMANOVA indicated a main effect of valence, *F*_(2, 62)_ = 4.97, *p* = 0.020, $\eta_{\text{p}}^{2}$ = 0.14, ε = 0.72, a main effect of source, *F*_(1, 31)_ = 9.70, *p* = 0.004, $\eta_{\text{p}}^{2}$ = 0.24, and a valence x source interaction effect, *F*_(2, 62)_ = 3.39, *p* = 0.040, $\eta_{\text{p}}^{2}$ = 0.10. The Bayes factors did not support the interaction model as the two main effects model gathered the most robust evidence against the null model (BF_10_ = 1888.860 ± 1.90%; 1888.860/935.928 = 2.02). The results indicate that participants rated self-referentially words as more memorable (*M* = 4.17, *SE* = 0.14) than words studied in the common condition (*M* = 3.94, *SE* = 0.12; *p* = 0.004, *d* = 0.55, 95% CI [0.08, 0.39]; see Table 1 and Figure 2D). The valence effect was inconclusive: Bayes factors only showed anecdotal evidence in favor of the valence model (BF_10_ = 1.029 ± 0.73%); the pairwise comparisons revealed marginally significant differences between the JOS ratings of positive words and both neutral (*p* = 0.074, *d* = 0.42, 95%CI [-0.01, 0.25]) and negative words (*p* = 0.064, *d* = 0.43, 95% CI [-0.01, 0.35]).

The metamnemonic judgments regarding source memory during encoding only partially complied with the pattern of results obtained in the recognition test. Although JOSs ratings indicated that self-referentially encoded words were regarded as more memorable than words studied in the common condition, which was in line with the self-reference benefit observed in the recognition test, the JOS ratings concerning valence were unclear. Additionally, the gamma correlations revealed no reliable association between source judgments and source recognition as the coefficients did not differ from zero, *t*_(31)_ ≤ 1.86, *p* ≥ 0.07. Furthermore, no significant differences were found between conditions (valence: *F*_(2, 54)_ = 0.10, *p* = 0.905, $\eta_{\text{p}}^{2}$ = 0.003; source: *F*_(1, 27)_ = 0.80, *p* = 0.378, $\eta_{\text{p}}^{2}$ = 0.03; interaction: *F*_(2, 54)_ = 0.09, *p* = 0.917, $\eta_{\text{p}}^{2}$ = 0.003), and the coefficients remained positive yet small (see Table 1). The null effects were supported by the Bayesian analysis (valence: BF_10_ = 0.058 ± 1.06%; source: BF_10_ = 0.287 ± 0.72%; interaction: 0.002/0.016 = 0.13). Thus, the results pointed to the lack of an association between source memory predictions and memory performance.

### Discussion: Experiment 2

By changing the encoding strategy from Experiment 1 to Experiment 2, we observed a differential influence of valence on internal source memory, item memory, and JOSs. As expected, a self-referential benefit on item memory was observed (Rogers et al., 1977; Kuiper and Rogers, 1979; Symons and Johnson, 1997), and this occurred irrespective of stimulus valence. The item memory performance was also enhanced for neutral relative to negative words (yet no significant differences were observed in the case of positive words), and this was observed in both encoding conditions. Thus, we partially replicated prior evidence showing that neutral stimuli encoded in a self-referential manner are better remembered than negative stimuli (Yang et al., 2012). However, the hypothesized difference between negative and positive stimuli (based on prior studies—Yang et al., 2012; Leshikar et al., 2015) was not observed. Notwithstanding, the EEM was not replicated in the common condition. The response bias also changed with the encoding strategy: in cases of uncertainty, participants showed an increased tendency to respond “new” for both negative and neutral stimuli relative to positive stimuli; participants also used a more conservative response criterion for stimuli encoded in the common condition than for stimuli encoded in the self-referential condition. On average, participants appeared to show a conservative to neutral response criterion (see Table 1). The only exception was observed in the case of self-referentially encoded positive words, whose mean Br was above 0.50. In the case of internal source memory performance, an interaction effect between valence and encoding strategy emerged, revealing that source recognition was improved for both neutral and positive words compared to negative stimuli in the self-reference task, which partially corroborates previous findings (e.g., Durbin et al., 2017; Zhang et al., 2018). A different pattern was observed in the common task, in which emotional words led to reduced source memory accuracy in contrast to neutral words. Specifically, valence impaired internal source memory, in agreement with prior studies (see Appendix). The JOSs ratings were also sensitive to self-referential processing as self-referentially encoded words were regarded as more memorable than words studied in the common condition. Regarding valence, the effect was less clear, which stands in contrast with Experiment 1 and previous studies (e.g., Zimmerman and Kelley, 2010; Tauber and Dunlosky, 2012; Hourihan et al., 2017). Nevertheless, no significant associations were found between the JOSs ratings and internal source memory performance.

## General Discussion

The main goal of this study was twofold: to explore how stimulus valence influences internal source monitoring, specifically when the encoding conditions are also characterized by memory enhancing features (production and self-reference effect); to specify the role played by stimulus valence in immediate prospective judgments concerning internal source memory (JOSs ratings). Overall, the results revealed that internal source monitoring, item memory, and JOSs were differently modulated by the encoding strategy, which was supported by participant-based results (RMANOVA), Bayes factors, and multinomial models (see Supplementary Material). Specifically, in Experiment 1, the effects of valence on item and source memory were not significant, even though the production effect was replicated for item memory. The JOSs ratings were sensitive to both valence and production mode. In turn, Experiment 2 revealed that the self-referential processing enhanced item and source memory differently as a function of valence. The JOSs ratings were also sensitive to the self-reference effect.

The current study supported the notion that self-referential conditions benefit both item and internal source memory (Hamami et al., 2011; Serbun et al., 2011; Leshikar and Duarte, 2012, 2014; Kalenzaga et al., 2015; Durbin et al., 2017; Yin et al., 2018; Zhang et al., 2018). Nonetheless, in the case of the production effect, the beneficial effects were only demonstrated for item memory but not for source memory, which stands in contrast with previous evidence (Ozubko et al., 2014). Specific methodological factors may have attenuated the production effect such as intentional encoding instructions, the use of the “do not know” option in the test phase (which may have led to a more conservative response in situations in which the participants were not able to recollect the source but they knew the words were familiar), or the source recognition measure that included the proportion of incorrect source attributions. Indeed, we observed that the proportion of incorrect source attributions for words that were read aloud was significantly higher than for words read silently. As participants believed that words read aloud were more memorable than words read silently (which was reflected in JOSs ratings), they may have been more prone to attribute a specific word read aloud to the other source when they only knew that a specific word was studied before. Nonetheless, a lack of effect was also expected, given that prior studies that probed the effects of valence on internal source memory also reported a null (e.g., Kensinger and Schacter, 2006a; Sharot and Yonelinas, 2008; Ferré et al., 2019, Experiment 1) or an impairment effect (e.g., Newsome et al., 2012; Otani et al., 2012a,b; Mao et al., 2015; Ferré et al., 2019, Experiment 2 and 3; see Appendix). Critically, both results were observed in the current study but with distinct encoding conditions, i.e., no effect in Experiment 1 and an impairment effect in the non-self-referential condition in Experiment 2. Furthermore, an enhancement effect emerged for both neutral and positive words studied in a self-referential manner.

Whereas an impairment or lack of effect might be explained by the object-based binding theory (Mather, 2007), as production mode and cognitive operations (self-referential or common judgments) can be deemed as extrinsic features of the item, this theoretical framework cannot account for the self-referential benefit of source memory in the case of neutral and positive words, especially considering that stimuli in Experiment 2 were matched for arousal. In this context, the arousal-biased competition theory (Mather and Sutherland, 2011) offers a more suitable framework for the source memory effects found in the current study, considering that both emotional and non-emotional information can be prioritized according to the current goals and motivations of the individual. Even though both positive and negative stimuli were more arousing than neutral stimuli in Experiment 1, the participants were instructed to effortfully encode both item and production mode. Hence, even if the items that were read aloud were more perceptually salient, all items were goal-relevant and likely to receive similar processing resources.

Considering that self-referential processing promotes a more organized, elaborative and efficient processing in comparison with other perceptual and semantic tasks, such as common judgments (which can be deemed as unusual tasks; Symons and Johnson, 1997; D'Argembeau et al., 2005), participants may have prioritized information that was relevant to describe themselves in Experiment 2. In fact, words that are perceived as self-descriptive tend to be better remembered than non-self-descriptive words (Kuiper and Rogers, 1979). Participants also tend to favor positive information and to disregard negative self-referential information (D'Argembeau et al., 2005; Watson et al., 2008; Zhang et al., 2018), a tendency that has been observed irrespective of age, gender, or cultural background (Mezulis et al., 2004). Moreover, the information that fits the current self-scheme may receive a deeper and elaborative processing, whereas non-fitting information may be processed in a shallow manner, which may result in a less successful memory performance (Kuiper and Rogers, 1979; D'Argembeau et al., 2005). Thus, enhanced source memory might be expected for both neutral and positive self-referential words as they are likely to match the current self-schema (the proportion of “yes” responses was also higher for positive and neutral words; see Supplementary Material). In contrast, the negative self-referential words may have been neglected as they are less likely to match the current self-schema, leading to a decline in source memory performance.

In the case of the common condition, a similar result to Experiment 1 could be expected, as identical intentional encoding conditions were required during the learning phase. However, an impairment effect was observed for emotional words encoded in the common condition. This finding might be accounted for the higher proportion of incorrect source attributions found for positive words in comparison with neutral words (see Supplementary Material). Although this represents a limitation of the current study, it might be the case that the emotional adjectives selected here were more prototypical self-descriptors than the neutral adjectives. Thereby, participants could be more likely to confound the source of emotional adjectives studied in the common condition. Likewise, the neutral adjectives could offer a more congruent match with the common task, resulting in a better source memory performance. According to the arousal-biased competition theory (Mather and Sutherland, 2011), the former explanations might be related to top-down processes such as the relevance of the stimuli to the current task. Additionally, they also fit with the source-monitoring account (Johnson et al., 1993), as decisions regarding the origin of an event are influenced not only by qualitative characteristics that take place during encoding (e.g., cognitive operations), but also by previous general knowledge and schemas (e.g., stereotypes; beliefs). Thus, when there are few qualitative features to distinguish between sources, which is the case of internal source memory decisions, participants might rely on their beliefs. In the test phase, if participants consider a specific word as a self-descriptor (which might be more likely to happen in the case of positive adjectives), they might be biased to select the self-reference option, especially when they cannot recall other qualitative features associated with the word, even when a “do not know option” is available. In fact, positive adjectives that were self-referentially encoded were also associated with a more liberal response criterion (see Table 1), suggesting that participants were biased to deem positive new items as “old”.

The subjective JOSs ratings were also informative of how sensitive the participants were to both valence and encoding strategy during the encoding phase. In Experiment 1, the source of both positive and negative words was regarded as more memorable than neutral words. This pattern has also been reported in studies using JOLs (e.g., Jönsson et al., 2005; Zimmerman and Kelley, 2010; Tauber and Dunlosky, 2012; Nomi et al., 2013; Hourihan and Bursey, 2017; Hourihan et al., 2017). Here, it was extended to JOSs. As the processing of emotional stimuli shows some advantages in comparison with neutral stimuli in terms of attention, organization, and distinctiveness (D'Argembeau and Van der Linden, 2004; Maddox et al., 2012; Talmi, 2013), these factors may also play a role in metamemory judgments. Furthermore, the source of words that were read aloud was rendered as more memorable than words read silently, following previous studies examining JOLs (Castel et al., 2013). Also, in Experiment 2, JOSs ratings revealed that self-referentially encoded words were judged as more memorable when compared to words studied in the common condition. Notwithstanding, the effect of valence was inconclusive, and our initial predictions were not confirmed as only marginal results supported the preference of the participants for positive information. Overall, both experiments demonstrate that participants were sensitive to the potential advantage of reading items aloud or processing them self-referentially. This finding might be accounted for the cue-utilization hypothesis (Koriat, 1997), which posits that prospective metamemory judgments can be modulated by specific characteristics that provide a sense of how easy/difficult it is to process and learn an item.

According to the cue-utilization hypothesis, JOLs are inferential in nature and their predictive value depends on whether the cues used to make metamemory judgments converge with variables affecting memory performance. Thus, in some instances immediate metamemory judgments might predict future memory performance (e.g., Mazzoni and Nelson, 1995; Carroll et al., 2001; Dutton and Carroll, 2001), whereas the same may not occur in other situations. For instance, in a previous study by Carroll et al. (1999), exploring JOLs and JOSs applied to reality monitoring decisions (seen vs. imagined), participants' predictions regarding source memory performance did not differ from chance. In the current study, this result is extended to internal source memory, specifically in Experiment 2 and in some conditions of Experiment 1. Although the predictions for some conditions of Experiment 1 were different from chance, no reliable differences in the gamma coefficients were found between encoding strategy and valence conditions. These findings stand in contrast with the findings of Dutton and Carroll (2001) but agree with the JOLs findings (Hourihan and Bursey, 2017; Hourihan et al., 2017). As suggested by Kelly et al. (2002), JOSs can be considered an unfamiliar task and, plausibly, participants might have based their JOSs on item memorability rather than source memory memorability. In fact, it has been previously suggested that JOLs and JOSs are associated and that similar cues might be used to make these predictions (Carroll et al., 1999, 2001; Kelly et al., 2002). As immediate prospective metamemory judgments are performed item by item, participants may rely their decisions on short-term memory and on other factors (e.g., the nature of the elaborative processes) that do not have enough diagnostic value to predict which information will be later remembered or forgotten.

The indirect analysis of item memory performance did not reveal an EEM effect, contrary to our initial prediction and several prior studies (e.g., Davidson et al., 2006; Kensinger and Schacter, 2006a; Maddock and Frein, 2009; Schmidt et al., 2011; Maddox et al., 2012; Yang et al., 2012; Schümann et al., 2018). Specifically, in Experiment 1, no difference was found when emotional words were compared to neutral words. In Experiment 2, neutral words were associated with higher recognition rates than negative words irrespective of the encoding strategy. Enhanced item memory for neutral compared to negative words encoded in the self-referential condition was expected (Yang et al., 2012). Nonetheless, a similar benefit was also expected for positive words that were processed self-referentially in relation to negative words (Yang et al., 2012; Leshikar et al., 2015), even though this prediction was not confirmed by the current study. A failure to observe such effect is rather in agreement with the studies of D'Argembeau et al. (2005, Experiment 2) and of Zhang et al. (2018, Experiment 2) that compared positive and negative traits. Even considering the consistency of the EEM effect in the literature (see Murphy and Isaacowitz, 2008 for a meta-analysis), other studies have also failed to report significant emotion effects on memory tasks (e.g., Newsome et al., 2012; Hourihan and Bursey, 2017; Ferré et al., 2019), particularly when using recognition tests (e.g., Doerksen and Shimamura, 2001; D'Argembeau and Van der Linden, 2004; Davidson et al., 2006) and short intervals between study and test (e.g., Mitchell et al., 2006; Sharot and Yonelinas, 2008; Yonelinas and Ritchey, 2015; Wang, 2018). The null effect might also be explained by the higher proneness to false alarms observed in the case of emotionally salient words, especially when they are intentionally studied (D'Argembeau and Van der Linden, 2004; Davidson et al., 2006, Experiment 2B; Cook et al., 2007; Sharot and Yonelinas, 2008). Indeed, this explanation could partially account for the item memory difference between neutral and negative words in Experiment 2, irrespective of the encoding task, as the false alarm rates were higher for emotional compared to neutral words (see Supplementary Material). Additionally, the negative adjectives studied in the self-referential condition were also associated with a greater proportion of misses compared to positive adjectives studied in the same condition (see Supplementary Material). This last finding might have also contributed to the statistically significant difference between negative and neutral stimuli, and for the intermediate position of positive stimuli, which did not differ from both neutral and negative stimuli.

Although the former interpretation seems to apply to Experiment 2, the same cannot be assumed for Experiment 1, especially because the false alarm rates did not differ across negative, neutral and positive words (see Supplementary Material). In this regard, the short interval between study and test may be an important factor to consider. Prior studies revealed that the relationship between the brain activity during encoding and the subsequent memory performance for neutral stimuli is stronger at a short interval than at a long interval, whereas the same relationship appears to be more stable over time in the case of emotional stimuli (Mickley Steinmetz et al., 2012). Moreover, emotional information seems to be more resistant to forgetting (Sharot and Phelps, 2004; Mitchell et al., 2006; Ritchey et al., 2008; Sharot and Yonelinas, 2008; Weymar et al., 2009, 2011; Schaefer et al., 2011; Yonelinas and Ritchey, 2015). As in Experiment 1 the study phase was immediately followed by the test phase, it could be the case that the EEM effect would emerge if longer study-test intervals were implemented (Sharot and Phelps, 2004; Mitchell et al., 2006; Sharot and Yonelinas, 2008; Schaefer et al., 2011; Yick et al., 2015), consistent with consolidation processes (Hamann, 2001; Talmi, 2013). However, previous studies also indicated that even in immediate conditions, negative arousing stimuli can be better recognized than neutral stimuli (e.g., Wirkner et al., 2018). Whereas in Experiment 2 stimuli were controlled for arousal, in Experiment 1 both negative and positive stimuli had higher arousal ratings than neutral ones (according to Soares et al., 2012). As such, a difference could be at least expected between negative and neutral stimuli in the context of Experiment 1. Nevertheless, we only observed a trend for a beneficial effect of negative information compared to positive information, which is in line with prior evidence showing that memory superiority effects are more consistent for negative information and less clear for positive information (e.g., Ochsner, 2000; Wang and Fu, 2011; Otani et al., 2012a,b; Rossi-Arnaud et al., 2018; Wang, 2018). In this context, differences in valence/arousal between the original ANEW ratings and the participants' ratings in the current study should be also considered. We cannot rule out the possibility that the findings of this experiment were somehow affected by a mismatch between the original ratings and the subjective evaluations of the words by the participants of the current study (see Davidson et al., 2006, Experiment 1 and 2A, and Koenig and Mecklinger, 2008 for examples).

Another plausible explanation for the lack of an EEM effect may relate to the experimental conditions as participants were aware that their memory would be tested later, and they were overtly instructed to memorize item and source. Thus, despite a processing advantage of emotional words over neutral words in terms of attention, distinctiveness, and organization (D'Argembeau and Van der Linden, 2004; Maddox et al., 2012; Talmi, 2013), the impact of these factors might have been attenuated by intentional learning. Hence, it is possible that valence effects were overshadowed by similar attentional resources and effortful encoding for all types of stimuli (e.g., Ferré et al., 2019, Experiment 1). When the relevance of the stimuli to the current goals is identical across emotional and neutral conditions, the memory performance may not differ as a function of valence and/or arousal (Ochsner, 2000), in line with the arousal-biased competition theory (Mather and Sutherland, 2011). Furthermore, experimental conditions relying on a recognition test instead of free recall (e.g., Doerksen and Shimamura, 2001; D'Argembeau and Van der Linden, 2004), the type of recognition test (e.g., direct source memory test instead of old/new judgments followed by a source memory test conditioned to old items), different study-test cycles with few trials (e.g., Adelman and Estes, 2013), and the short period between study and test (e.g., Sharot and Phelps, 2004; Mitchell et al., 2006; Sharot and Yonelinas, 2008; Wang, 2018), might have also contributed to the current pattern of findings.

### Limitations

The manipulation of valence was critical in the current study, but the full control of stimulus arousal was not possible in the case of Experiment 1. Therefore, it is not possible to clearly dissociate valence from arousal effects in this case. Moreover, word selection relied on norms considering the combined ratings of male and female participants, yet the sample was mainly composed of female participants. Considering prior evidence for sex differences in the encoding and recall of emotional events (e.g., Galli et al., 2011; Glaser et al., 2012), this may limit the generalization of the current findings. Also, the role of specific stimulus properties (e.g., familiarity; concreteness; imageability; age of acquisition; self-descriptive/commonness characteristics; Adelman and Estes, 2013; Fan et al., 2016) was not accounted for in this study. Another limitation concerns the lack of control of factors such as attention, organization, distinctiveness, or personal motivations, which may impact upon the emotion-memory dynamics (Talmi, 2013), and are critical for a more thorough discussion of theoretical frameworks such as the object-based binding theory (Mather, 2007) and the arousal-biased competition theory (Mather and Sutherland, 2011). Likewise, it was not possible to clarify whether the results observed in the test phase depended on mechanisms that operated during encoding and/or recognition (see Hamann, 2001, and Levine and Edelstein, 2009, for reviews), even if the experimental manipulations were restricted to the encoding phase and even if the influence of consolidation processes is not expected in conditions of immediate recognition.

## Conclusion

The current study confirmed the role of task-related factors in the interplay between emotion and memory. Specifically, emotional stimuli encoded in different conditions (read aloud vs. silently; self-reference vs. common) led to distinct findings in terms of internal source memory, item memory, and JOSs ratings. In line with the source-monitoring framework, we showed that emotional events do not always enhance episodic memory recognition, and that their impact is not the same for source and item memory (Johnson et al., 1993; Jurica and Shimamura, 1999). Although the encoding strategies tested here—the production and the self-reference effect—are known to benefit item memory, a similar benefit was only found for internal source memory in the case of positive and neutral self-referenced words. Further research is needed to clarify the relationship between emotion and source memory, considering that stimulus type (e.g., Durbin et al., 2017), the type of source discrimination (e.g., Boywitt, 2015), and the encoding strategy (e.g., Kuhlmann and Touron, 2012, 2017) may affect this relationship. The same applies to JOSs ratings, which were sensitive to both valence and encoding strategy manipulations, although their predictive value was not confirmed. Together, the current experiments demonstrate how different encoding strategies modulate the effects of valence on distinct features of episodic memory, and on prospective metamemory judgments. Future studies should account for the way information is encoded when probing how emotion influences different facets of episodic memory.

## Ethics Statement

All subjects gave written informed consent in accordance with the Declaration of Helsinki. This study was approved by the local Ethics Committee (Subcomissão de Ética para as Ciências da Vida e da Saúde, Universidade do Minho, Braga, Portugal; SECVS 105/2016).

## Author Contributions

DP conceived the study concept and design under the supervision of AS and AP. DP performed data collection, analysis and interpretation, and drafted the first version of the manuscript. All authors provided a critical review of the manuscript and approved its final version.

### Conflict of Interest Statement

The authors declare that the research was conducted in the absence of any commercial or financial relationships that could be construed as a potential conflict of interest.
