# Supplementary material for: Differential Effects of Valence and Encoding Strategy on Internal Source Memory and Judgments of Source: Exploring the Production and the Self-Reference Effect
Source: Front Psychol. 2019 Jun 12;10:1326. doi: 10.3389/fpsyg.2019.01326 (PMC6582403; doi:10.3389/fpsyg.2019.01326)
Supplement: Supplementary file 1 [file Data_Sheet_1.docx]

**Appendix**

*Summary of Findings Reported in Studies Probing the Effects of Stimulus Emotional Properties on Source Memory Performance, Considering External, Internal, and Reality Monitoring Tasks*

| Study | | | *N* (m/f; *M*_age_) | Type of stimuli | Valence and/or arousal properties: *M* | Encoding task (intentional/incidental) | SM manipulation | Type of SM test | SM effect (effect size range) |  |
| --- | --- | --- | --- | --- | --- | --- | --- | --- | --- | --- |
| **External SM** | | | | | | | | | |  |
| Doerksen and Shimamura, 2001 | | E1 | 24 (16f; 19.6) | 128 words | 32 Pos: V ≥ 5.25  32 Neg: V ≤ 2.5  64 Neu: n/a | Read word silently (intentional) | Stimuli color | Recognition test | ↑ Pos, Neg |  |
|  |  | E2 | 24 (17f; 19.9) | = E1 | = E1 | = E1 | Color of the border | = E1 | ↑ Pos, Neg |  |
| Kensinger and Corkin, 2003 | | E2 | 18 (18m; 22.7) | 280 words (ANEW) | 140 Neg: set A (V = 1.78; A = 6.95); set B (V = 1.91; A = 7.16)  140 Neu: set A (V = 5.83; A = 3.12); set B (V = 5.65; A = 3.35) | Rate each word as abstract or concrete (intentional) | Stimuli color | Recognition test | ↑ Neg |  |
|  |  | E4 | 20 (20m; 21.5) | 180 words | 60 taboo: V = 3.58; A = 8.02  60 Neg: V = 1.78; A = 3.35  60 Neu: V = 5.22; A = 4.51 | = E2 | = E2 | = E2 | ↑ Neg  (↑ taboo) |  |
| D'Argembeau and Van der Linden, 2004 | | E1 | 32 (12f; 22.9) | 96 words | 24 Pos: V ≥ 4.10  24 Neg: V ≤ 1.90  48 Neu: 2.70 ≤ V ≥ 3.30 | Read word (16 participants intentional; 16 incidental) | Stimuli color (two colors) | Recognition test | 🗙 |  |
|  |  | E2 | 32 (22f; 21.8) | 64 words | 16 Pos: n/a  16 Neg: n/a  32 Neu: n/a | Read word (intentional) | = E1 | = E1 | 🗙  (continued) |  |
| Study | | | *N* (m/f; *M*_age_) | Type of stimuli | Valence and/or arousal properties: *M* | Encoding task (intentional/incidental) | SM manipulation | Type of SM test | SM effect (effect size range) |  |
|  | | E3 | 48 (34f; 21.5) | = E1 | = E1 | = E1 | = E1 (four colors) | = E1 | ↑ Pos, Neg (incidental)  🗙 (intentional) |  |
|  |  | E4 | 48 (34f; 22.5) | = E1 | = E1 | = E1 | Spatial location | = E1 | ↑ Pos, Neg (both incidental and intentional) |  |
| D'Argembeau and Van der Linden, 2005 | | | 48 (24f; 23.7) | 126 pictures (IAPS) | 42 Neg: V = 2.73; A = 5.69  42 Neu: V = 4.98; A = 3.15  42 Pos: V = 7.40; A = 5.39 | Look at pictures to recognize them later (intentional for the item, not for the source) | Temporal context (list 1 vs. list 2 vs. list 3) | Recognition test | ↑ Neg (.38 < *d* < .58) |  |
| Davidson et al., 2006 | | E1 | 32 (n/a; 21.6) only YA | 48 words (ANEW) | 24 Emotional: n/a  24 Neu: n/a | Indicate weather each word was emotional or neutral (incidental) | Voice (male vs. female) | Recognition test (two-alternative forced choice) | ↑ Emotional (if normative ratings)  🗙 (if participants’ ratings) |  |
|  | | E2A | 32 (n/a; 18.5) | = E1 | = E1 | = E1 | Stimuli color (two colors) | = E1 | 🗙 (participants’ ratings) |  |
|  | | E2B | 33 (n/a; 18.3) | = E1 | = E1 | = E1 (intentional) | = E2A | = E2A | 🗙 |  |
| Mitchell et al., 2006 | | | 19 (10f; 20.6) | 360 pictures (266 IAPS + other sources) | 180 Neg: V = 2.63; A = 6.15  180 Neu: V = 6.44; A = 3.67 | Memorize picture and location (intentional) | Spatial location (six locations) | Recognition test | ↓ Neg (short-term memory) |  |
| Koening and Mecklinger, 2008 | | | 20 (10 f; 21) | 480 pictures (IAPS) | 160 Pos: V = 7.13; A = 5.04  160 Neg: V = 2.97; A = 5.20  160 Neu: V = 5.09; A = 3.43 | Respond if indoors or outdoors (intentional) | Temporal context (list 1 vs. list 2) and spatial location (booth 1 vs. booth 2) | Recognition test | 🗙  (continued) |  |
| Study | | | *N* (m/f; *M*_age_) | Type of stimuli | Valence and/or arousal properties: *M* | Encoding task (intentional/incidental) | SM manipulation | Type of SM test | SM effect (effect size range) |  |
| Maddock and Frein, 2009 | | E1A | 48 (34f; 19.77) | 36 words | 12 Pos: V = 2.74; A = 6.98  12 Neg: V = 3.04; A = 7.13  12 Neu: V = 0.26; A = 2.57 | Rate pleasantness (incidental) | Spatial location (6 x 7 cell spatial grid) | Free recall | ↓ Neg |  |
|  | | E1B | 48 (40f; 19.08) | 36 words | 12 Pos: V = 2.36; A = 6.67  12 Neg: V = 2.62; A = 6.78  12 Neu: V = 0.22; A = 2.62 | = E1A | = E1A | = E1A | ↓ Neg |  |
|  |  | E3 | 48 (29f; 19.9) | = E1A | = E1A | = E1A | Temporal context (which word appeared first in word pairs) | = E1A | ↓ Neg |  |
|  | | E4 | 24 (17f; 20.67) | 72 words (E1A + E1B) | = E1A + E1B | = E1A (intentional) | = E1A (2 x 2 cell spatial grid) | Recognition test | ↓ Neg |  |
| Rimmele et al., 2011 | | E1 | 25 (13f; 22.58) | 120 scenes (IAPS) | 60 Neg: V = 2.88; A = 5.62  60 Neu: V = 5.58; A = 3.87 | Judge if the frame color appeared in the scene (incidental) | Color of the border | Recognition test | ↓ Neg |  |
|  | | E2 | 26 (16f; 24.35) | = E1 | = E1 | = E1 | Spatial location (four locations) | = E1 | ↑ Neg |  |
|  | | E3 | 32 (18f; 23.90) | = E1 | = E1 | = E1 | Temporal context (first vs. second vs. third block) | = E1 | ↑ Neg |  |
|  | |  |  |  |  |  |  |  |  |  |
|  | |  |  |  |  |  |  |  |  |  |
|  | |  |  |  |  |  |  |  |  |  |
|  | |  |  |  |  |  |  |  |  |  |
|  | |  |  |  |  |  |  |  | (continued) |  |
| Study | |  | *N* (m/f; *M*_age_) | Type of stimuli | Valence and/or arousal properties: *M* | Encoding task (intentional/incidental) | SM manipulation | Type of SM test | SM effect (effect size range) |  |
| Schmidt et al., 2011 | | E1 | 24 (14f; 19.7) | 540 pictures (IAPS) | Pos: V = 6.98  Neg: V = 3.06  HA: A = 5.92  LA: A = 4.19  Neu: V = 5.08; A = 3.10 | Living judgment (something alive) or common judgment (something that might be encounter within a typical month) or approach judgment (move closer in “real life”) (incidental) | Temporal context (first vs. second vs. third list)  Spatial location (left vs. center vs. right) | Recognition test | ↑ HA (compared to LA) (η^2^_p_ = .17)  🗙 (valence)  (for R judgments) |  |
|  | | E2 | 24 (13f; 19.7) | n/a | n/a | Memorize objects and background scenes  (intentional) | Order (first vs. second vs. third in the order of appearance)  Spatial location (3 x 3 grid) | Recognition test | ↑ HA (compared to LA) (η^2^_p_ = .24)  🗙 (valence) |  |
|  | |  |  |  |  |  |  |  |  |  |
| Wang and Fu, 2011 | | | 136 (86f; 22.91) | 120 Chinese words | 20 Pos: set 1 (V = 6.31; A = 6.02); set 2 (V = 6.42; A = 6.01)  20 Neg: set 1 (V = 2.44; A = 5.77); set 2 (V = 2.42; A = 5.79)  20 Neu: set 1 (V = 5.06; A = 4.97); set 2 (V = 5.05; A = 4.97) | Memorize words (intentional) | Stimuli color | Recognition test | 🗙 (for different delay conditions: immediate; 19 min; 63 min; 4.9 h; 8.75h; 1 day; 6 days; 14 days) (.004 < η^2^_p_ < .051) |  |
| Rimmele et al., 2012 | | E1 | 25 (13f; 22.52) | 120 scenes (IAPS) | 60 Neg: V = 2.88; A = 5.62  60 Neu: V = 5.58; A = 3.87 | Judge if the color of dots appeared elsewhere in the scene | Color of dots presented in the scene | Recognition test | ↓ Neg  (*d* = .84)  (continued) |  |
| Study | |  | *N* (m/f; *M*_age_) | Type of stimuli | Valence and/or arousal properties: *M* | Encoding task (intentional/incidental) | SM manipulation | Type of SM test | SM effect (effect size range) |  |
| Boywitt, 2015  (continued) | | E1 | 101 (84%f; n/a) | 120 pictures (IAPS) | LA: V = 5.28; A = 2.88  MA: V = 5.10; A = 4.82  HA: V = 4.55; A = 6.76 | Memorize pictures (intentional) | Color of the border | Recognition test | 🗙 (K judgments) (η^2^_p_ = .028)  ↓ Increasing arousal (R judgments) (η^2^_p_ = .26) |  |
|  | | E2 | 83 (77%; n/a) | = E1 | = E1 | = E1 | Spatial location (left vs. right) | = E1 | ∩ (R and K) (η^2^_p_ = .139) |  |
| MacKenzie et al., 2015 | | E1 | 32 (23f; 19) | 60 pictures (IAPS) | 20 Pos: V = 7.92; A = 4.53  20 Neg: V = 2.74; A = 4.53  20 Neu: V = 5.27; A = 4.53 | Memorize picture and border color (intentional) | Color of the border | Recognition test | ↓ Pos, Neg |  |
|  |  | E2 | 32 (20f; 21) | = E1 | = E1 | Memorize picture and color (intentional) | Stimuli color | = E1 | ↓ Neg |  |
| Yick et al., 2015 | | | 42 (28f; 22) | 720 pictures (IAPS + Google image) | Emotional: V = 1.98; A = 3.21  Neu: V = 3.21; A = 1.95 | Rate emotionality (intentional) | Temporal context (block 1 vs. block 2) | Recognition test | ↑ Emotional pictures |  |
| **Reality monitoring** | | | | | | | | | |  |
| Kensinger and Schacter, 2006b | | E1 | 32 (18f; 21.3) | 128 words (ANEW) | 64 Neg: V = 3.00; A = 6.2  64 Neu: V = 5.80; A = 3.9 | Verify if the height of the first letter was lower than the height of the fourth letter (half of the participants incidental and other half intentional) | Heard/imagined vs. seen | Recognition test | ↑ Neg (both incidental and intentional) (η^2^_p_ = .45) |  |
|  | |  |  |  |  |  |  |  |  |  |
|  | |  |  |  |  |  |  |  | (continued) |  |
| Study | |  | *N* (m/f; *M*_age_) | Type of stimuli | Valence and/or arousal properties: *M* | Encoding task (intentional/incidental) | SM manipulation | Type of SM test | SM effect (effect size range) |  |
|  | | E2 | 20 (12f; 21.5) | 450 words + 450 pictures of objects | HA: words (A = 2.96); pictures (A = 3.07)  LA: words (A = 0.59); pictures (A = 0.67) | Indicate if each object was bigger or smaller than a shoebox (incidental) | Imagined vs. seen | Recognition test | ↑ HA (η^2^_p_ = .33) |  |
|  |  | E3A | 16 (8f; 21.9) | = E1 | = E1 | = E1 (incidental) | = E1 | = E1 | ↑ Neg (η^2^_p_ = .62) |  |
|  | | E3B | 16 (n/a; 21.3) | = E2 | = E2 | = E2 (incidental) | = E2 | = E2 | ↑ HA (η^2^_p_ = .80) |  |
| Cook et al., 2007 | | E1 | 34 (n/a) | 270 words (ANEW) | 90 Pos: V = 7.70; A = 5.71  90 Neg: V = 2.31; A = 5.89  90 Neu: V = 5.18; A = 5.70 | Study the words  (incidental) | Heard/imagined vs. seen | Recognition test | ↓ Neg (only in comparison with Neu, not Pos) |  |
|  |  | E2 | 120 (n/a) | = 90 | = E1 (but only neg and neu words) | = E1 | = E1 | = E1 | ↓ Neg (for both mixed and pure lists) |  |
|  |  | E3 | 106 (n/a) | = 90 | = E1 (but only pos and neu) | = E1 | = E1 | = E1 | ↓ Pos (for both mixed and pure lists) |  |
|  | | E4 | 68 (n/a) | 160 categorial words representing love, joy, fear, sadness, and anger (and also neutral condition) | 128 emotional  32 neutral | Solving an anagram or rate the frequency of encounter in the last two weeks (incidental) | = E1 | = E1 | ↓ Pos, Neg |  |
|  | |  |  |  |  |  |  |  |  |  |
|  | |  |  |  |  |  |  |  | (continued) |  |
| Study | |  | *N* (m/f; *M*_age_) | Type of stimuli | Valence and/or arousal properties: *M* | Encoding task (intentional/incidental) | SM manipulation | Type of SM test | SM effect (effect size range) |  |
| McKague et al., 2012 | | | 133 (98f; 19.3); only 20 scoring lowest in the auditory-verbal hallucination questionnaire | 192 words (ANEW) | 96 Pos: V = 7.48; A = 5.44  96 Neg: V = 2.63; A = 5.60 | Generate aloud or heard sentences including a target word | Self-generated vs. other-generated | Recognition test | ↓ Neg (in comparison with Pos) (η^2^_p_ = .19) |  |
| Le Bigot et al., 2018 | E1 | | 42 (38f; 20.86) | 104 words (ANEW) | 52 Neg: V = 2.28; A = 5.20  52 Neu: V = 4.93; A = 3.36 | Produce a sentence out loud containing a specific word or hear another participant producing a sentence (incidental) | Self-generated vs. partner-generated | Free recall | ↓ Neg |  |
|  | E2 | | 46 (46f; 21.28) | = E1 | 52 Pos: V = 7.89; A = 6.36  Neu = E1 | = E1 | = E1 | = E1 | ↓ Pos |  |
| **Internal SM** | | | | | | | | | |  |
|  | | |  |  |  |  |  |  |  |  |
| Kensinger and Schacter, 2006a | | | 21 (11m; 18-35) | 360 words  360 pictures | 120 Pos: n/a  120 Neg; n/a  120 Neu: n/a | Animate judgment (something animate) or common judgment (something encountered in a typical month)  (incidental) | Animate judgment vs. common judgment | Recognition test | 🗙 |  |
| Cook et al., 2007 | | E4 | 68 (n/a) | 160 categorial words representing love, joy, fear, sadness, and anger (and also neutral condition) | 128 emotional  32 neutral | Solving an anagram or rate the frequency of encounter in the last two weeks (incidental) | Solving an anagram vs. rate the frequency of encounter in the last two weeks | Recognition test | ↓ Pos, Neg  (continued) |  |
| Study | | | *N* (m/f; *M*_age_) | Type of stimuli | Valence and/or arousal properties: *M* | Encoding task (intentional/incidental) | SM manipulation | Type of SM test | SM effect (effect size range) |  |
| Sharot and Yonelinas, 2008 | | | 24 (n/a; 18-22) | 360 photos (IAPS) | 180 Neg: V = 7.69; A = 6.79  180 Neu: V = 3.75; A = 3.03 | Rate visual complexity of the photo (incidental) | Colour task (colours of the photo to judge the complexity) vs. detail task (details of the photo to judge the complexity | Recognition test | 🗙 (for different delay conditions: 5 min; 24h) |  |
| Newsome et al., 2012 | | | 16 YA (8f; 25)  14 OA (5f; 67.7) | 432 pictures (IAPS) + 450 pictures (Google) | Pos: YA (V = 1.69; A = 2.50); OA (V = 1.59; A = 2.78)  Neg: YA (V = 4.25; A = 3.65); OA (V = 4.22; A = 4.04)  Neu: YA (V = 2.74; A = 2.12); OA (V = 2.77; A = 2.50) | Common judgment (something that might be seen within a typical month) or indoors judgment (the image depicts something indoor) (incidental) | Common judgment vs. indoors judgment | Recognition test | ↓ Neg |  |
| Otani, Jaffa, et al., 2012 | | | 40 (29f; n/a) | 60 pictures (IAPS) | 20 Pos: set A (V = 5.52; A = 4.07); set B (V = 6.31; A = 4.39)  20 Neg: set A (V = 2.10; A = 3.88); set B (V = 1.75; A = 4.65)  20 Neu: set A (V = 4.41; A = 2.34); set B (V = 4.27; A = 2.39) | Memorize the pictures and if apple- or orange-kind (intentional) | Apple-kind vs. orange-kind | Free recall | ↓ Neg (more misattribution errors than Neu pictures, yet no difference in relation to Pos pictures) (η^2^_p_ = .08) |  |
|  | | |  |  |  |  |  |  |  |  |
|  | | |  |  |  |  |  |  |  |  |
|  | | |  |  |  |  |  |  | (continued) |  |
| Study | | | *N* (m/f; *M*_age_) | Type of stimuli | Valence and/or arousal properties: *M* | Encoding task (intentional/incidental) | SM manipulation | Type of SM test | SM effect (effect size range) |  |
| Otani, Libkuman, et al., 2012 | | | 40 (26f; n/a) | 60 pictures (IAPS) | 20 Pos: set 1 (V = 7.11; A = 6.34); set 2 (V = 7.40; A = 6.26)  20 Neg: set 1 (V = 2.45; A = 6.02); set 2 (V = 2.17; A = 6.24)  20 Neu: set 1 (V = 4.99; A = 2.84); set 2 (V = 4.92; A = 2.75) | Remember pictures that were followed by a RRRR instruction slide (intentional) | To remember vs. to forget items | Free recall | ↓ Neg (more misattribution errors than Neu pictures, yet no difference in relation to Pos pictures) (η^2^_p_ = .11) |  |
| Leshikar et al., 2015 | | E1 | 24 YA (14f; 21.25)  24 OA (14f; 66.83) | 288 adjectives (ANEW) | 144 Neg: n/a  144 Pos: n/a | Self-referential judgment (self-descriptive) or common judgment (commonly used word) (intentional) | Self-referential judgement vs. common judgment | Recognition test | ↑ Pos (both age groups in the self-referential condition) |  |
| Mao et al., 2015 | | | 17 (10f; 23.4) | 1080 pictures (IAPS + CAPS) | 360 Pos: V = 2.12; A = 3.04  360 Neg: V = 3.90; A = 3.52  360 Neu: V = 2.84; A = 2.62 | Respond if the item belongs to the scene or people category (n/a) | Scenes vs. people | Recognition test | ↓ Neg, Pos (only for R judgments, not K judgments) (η^2^_p_ = .25) |  |
| Durbin et al., 2017 | | E1 | 24 (12f; 20.6) | 432 words (ANEW) | 144 Pos: V = 7.41; A = 5.68  144 Neg: V = 2.79; A = 5.70  144 Neu: V = 5.10; A = 3.84 | Self-referential judgment (degree of personal relevance) or story judgment (likelihood of appearance in a current news story) (n/a) | Self-referential judgement vs. story judgment | Recognition test | ↑ Pos (only for words encoded self-referentially compared to the story judgment) (η^2^_p_ = .16) |  |
|  | |  |  |  |  |  |  |  |  |  |
|  | |  |  |  |  |  |  |  | (continued) |  |
| Study | |  | *N* (m/f; *M*_age_) | Type of stimuli | Valence and/or arousal properties: *M* | Encoding task (intentional/incidental) | SM manipulation | Type of SM test | SM effect (effect size range) |  |
|  | | E2 | 24 (12f; 20.5) | 504 pictures (IAPS) | 168 Pos: V = 7.07; A = 5.28  168 Neg: V = 2.90; A = 5.34  168 Neu: V = 5.05; A = 3.36 | Self-referential judgment (= E1) or quality judgment (perceptual quality of the picture) | Self-referential judgement vs. quality judgment | Recognition test | ↑ Neg, Neu (for pictures encoded non-self-referentially than self-referentially) (.17 < η^2^_p_ < .30) |  |
| Zhang et al., 2018 | | E1 | 39 (27f; 20.2) | 80 trait words (pool of Chinese personality-trait adjectives) | 40 Pos: n/a  40 Neg: n/a | Self-referential judgment (indicate whether the word could be used to describe themselves) or other-referential judgment (indicate whether the word could be used to describe others) (incidental) | Self-referential judgment vs. other-referential judgment | Recognition test | ↓ Neg (in comparison with Pos in the self-referential condition)  ↑ Pos (for self-referent than other-referent words) (η^2^ = .34) |  |
|  | | E2 | 70 (54f; 20.6) | 160 trait words (pool of Chinese personality-trait adjectives) | 80 Pos: n/a  80 Neg: n/a | = E1 | = E1 | = E1 | ↓ Neg (in comparison with Pos in the self-referential condition)  ↓ Neg (for self-referential than other-referential condition) |  |
|  | |  |  |  |  |  |  |  | (continued) |  |
| Study | | | *N* (m/f; *M*_age_) | Type of stimuli | Valence and/or arousal properties: *M* | Encoding task (intentional/incidental) | SM manipulation | Type of SM test | SM effect (effect size range) |  |
| Ferré et al., 2019 | | E1 | 30 (23f; 22.3) | 120 words (emoFinder search engine; mostly ANEW) | 60 Neg: study set (V = 2.24; A = 6.53); test set (V = 2.21; A = 6.56)  60 Neu: study set (V = 5.13; A = 4.13); test set (V = 5.11; A = 4.08) | Memorize the words and the language of appearance (intentional) | Spanish vs. Catalan | Recognition test | 🗙 |  |
|  | | E2 | 30 (28f; 21.4) | = E1 | = E1 | Read aloud task (incidental) | = E1 | Recognition test | ↓ Neg  (.19 < η^2^_p_ < .33) |  |
|  | | E3 | 30 (20f; 24.3) | = E1 | 60 Pos: study set (V = 7.46; A = 6.02); test set (V = 7.48; A = 6.17)  Neu = E1 | = E1 | = E1 | Recognition test | ↓ Pos  (η^2^_p =_ .26) |  |

*Note*. A = Arousal; ANEW = Affective Norms for English Words; CAPS = Chinese Affective Picture Set; E = Experiment; HA = High Arousal; IAPS = International Affective Picture System; K = Know; LA = Low Arousal; MA = Medium Arousal; Neg = Negative; Neu = Neutral; n/a = Not Available; OA = Old Adults; Pos = Positive; R = Remember; SM = Source Memory; V = Valence; YA = Young Adults; ↑ = Enhancement Effect on Source Memory; ↓ = Impairment Effect on Source Memory; 🗙 = No Difference Between Emotional and Non-emotional stimuli on Source Memory; ∩ = Inverted “U” Relationship.
