# Supplementary material for: Differential Effects of Valence and Encoding Strategy on Internal Source Memory and Judgments of Source: Exploring the Production and the Self-Reference Effect
Source: Front Psychol. 2019 Jun 12;10:1326. doi: 10.3389/fpsyg.2019.01326 (PMC6582403; doi:10.3389/fpsyg.2019.01326)
Supplement: Supplementary file 2 [file Table_1.pdf]

## Supporting Information

Table S1

*Affective and Psycholinguistic Properties of the Stimuli Selected for Both Experiments*

| | | Valence category | | | *F*, *p* |
|---|---|---|---|---|---|
| | | Negative<br>*M* (*SD*) | Neutral<br>*M* (*SD*) | Positive<br>*M* (*SD*) | |
| Experiment 1 | Valence | 2.73 (0.42) | 5.07 (0.27) | 7.16 (0.43) | 2013.09, < .001 |
| | Arousal | 5.43 (0.53) | 4.01 (0.65) | 5.14 (0.94) | 63.62, < .001* |
| | Frequency | 18.67 (23.99) | 18.90 (16.80) | 23.43 (15.81) | 1.17, .31 |
| | N of letters | 7.12 (1.83) | 6.83 (1.72) | 7.30 (2.01) | 0.96, .39 |
| | N of syllables | 3.07 (0.90) | 2.92 (0.79) | 3.22 (0.94) | 1.75, .18 |
| Experiment 2 | Valence | 2.88 (0.40) | 5.06 (0.66) | 7.37 (0.38) | 978.29, < .001 |
| | Arousal | 4.83 (0.61) | 4.45 (0.78) | 4.73 (1.06) | 2.55, .82 |
| | Frequency | 14.54 (25.64) | 20.41 (24.11) | 16.40 (18.28) | 0.825, .44 |
| | N of letters | 7.52 (2.04) | 7.38 (2.10) | 7.88 (2.19) | 0.71, .49 |
| | N of syllables | 3.40 (0.89) | 3.23 (0.93) | 3.58 (0.99) | 1.72, .18 |

*Note*. * Post-hoc comparisons revealed that the arousal ratings for the neutral stimuli were lower than both negative and positive stimuli in Experiment 1.

Table S2

*Response Frequencies for Each Valence Condition in Experiment 1*

| | Negative | | | | Neutral | | | | Positive | | | |
|---|---|---|---|---|---|---|---|---|---|---|---|---|
| | Aloud | Silent | DNK | New | Aloud | Silent | DNK | New | Aloud | Silent | DNK | New |
| Aloud | 255 | 60 | 77 | 28 | 223 | 48 | 93 | 46 | 243 | 48 | 90 | 39 |
| Silent | 21 | 249 | 99 | 51 | 19 | 239 | 90 | 72 | 23 | 230 | 94 | 73 |
| New | 11 | 17 | 37 | 775 | 1 | 15 | 44 | 778 | 2 | 24 | 63 | 751 |

*Note*. DNK = Do Not Know.

Table S3

*Response Frequencies for each Valence Condition in Experiment 2*

| | Negative | | | | Neutral | | | | Positive | | | |
|---|---|---|---|---|---|---|---|---|---|---|---|---|
| | SR | Common | DNK | New | SR | Common | DNK | New | SR | Common | DNK | New |
| SR | 244 | 29 | 52 | 59 | 292 | 18 | 32 | 42 | 302 | 24 | 32 | 26 |
| Common | 48 | 164 | 74 | 97 | 33 | 206 | 66 | 79 | 69 | 152 | 81 | 81 |
| New | 16 | 16 | 59 | 672 | 10 | 11 | 47 | 699 | 16 | 19 | 76 | 657 |

*Note*. DNK = Do Not Know; SR = Self-Reference.

Table S4

*Multinomial Model Parameters for Item and Source Memory for each Experimental Condition (Source x Valence) in Experiments 1 and 2*

| | Experiment 1 – *M* (*SD*) | | | | | | Experiment 2 – *M* (*SD*) | | | | | |
|---|---|---|---|---|---|---|---|---|---|---|---|---|
| | Aloud | | | Silent | | | Self | | | Common | | |
| | Negative | Neutral | Positive | Negative | Neutral | Positive | Negative | Neutral | Positive | Negative | Neutral | Positive |
| D | .928 | .882 | .896 | .868 | .815 | .806 | .826 | .880 | .921 | .712 | .774 | .753 |
| d | .602 | .590 | .604 | .508 | .566 | .523 | .681 | .822 | .790 | .471 | .599 | .355 |
| a/g | .129 | .094 | .100 | .346 | .292 | .301 | .258 | .221 | .289 | .225 | .220 | .248 |

| | Experiment 1 | | | Experiment 2 | | |
|---|---|---|---|---|---|---|
| | Negative | Neutral | Positive | Negative | Neutral | Positive |
| a/g DK | .525 | .614 | .615 | .518 | .560 | .507 |
| b | .077 | .072 | .106 | .119 | .089 | .145 |

*Note*. DNK = Do Not Know.

Table S5

*Descriptive Statistics of Confidence Ratings and Response Time (JOSs Ratings, Source Correct Judgments, Correct Rejections) in each Experimental Condition (Task x Emotion) of Experiments 1 and 2*

| | Experiment 1 – *M* (*SD*) | | | | | | Experiment 2 – *M* (*SD*) | | | | | |
|---|---|---|---|---|---|---|---|---|---|---|---|---|
| | Aloud | | | Silent | | | Self | | | Common | | |
| | Negative | Neutral | Positive | Negative | Neutral | Positive | Negative | Neutral | Positive | Negative | Neutral | Positive |
| Confidence | 5.38 | 5.41 | 5.22 | 5.09 | 5.03 | 5.08 | 5.27 | 5.42 | 5.30 | 4.91 | 5.16 | 4.80 |
| source correct | (0.56) | (0.39) | (0.53) | (0.57) | (0.74) | (0.68) | (0.77) | (0.76) | (0.85) | (0.96) | (0.85) | (1.11) |
| RT JOSs | 1715 | 1650 | 1646 | 1876 | 1768 | 1763 | 1876 | 1902 | 1592 | 1782 | 1812 | 1668 |
| ratings | (868) | (827) | (886) | (1151) | (932) | (1185) | (718) | (762) | (574) | (766) | (935) | (679) |
| RT source | 3230 | 3007 | 3535 | 3558 | 3455 | 3909 | 3245 | 2827 | 2888 | 3600 | 3471 | 3488 |
| correct | (1076) | (835) | (1598) | (990) | (958) | (1291) | (1282) | (829) | (746) | (1150) | (1062) | (1187) |

| | Negative | Neutral | Positive | Negative | Neutral | Positive |
|---|---|---|---|---|---|---|
| Confidence CR | 5.06 (0.75) | 5.03 (0.76) | 4.75 (0.85) | 4.80 (0.86) | 4.96 (0.81) | 4.63 (0.92) |
| RT CR | 2092 (354) | 2039 (472) | 2319 (608) | 2278 (621) | 2131 (533) | 2373 (659) |

*Note*. CR = Correct Rejections; JOSs = Judgments of Source; RT = Response Time.

Table S6

*Results from the Pair-by-Pair Wilcoxon Tests Applied to the Proportion of Source Incorrect Responses, Do Not Know Responses, and Misses for Experiment 1 and Experiment 2*

| | Experiment 1 | | | | | Experiment 2 | | | | |
|---|---|---|---|---|---|---|---|---|---|---|
| | *n* | *T* | *z* | *r* | *p* | *n* | *T* | *z* | *r* | *p* |
| | Aloud | | | | | Self | | | | |
| Source incorrect | | | | | | | | | | |
| Negative - Neutral | 28 | 61 | - 1.10 | - .15 | .272 | 32 | 44 | - 1.86 | - .23 | .063 |
| Negative - Positive | 28 | 65 | - 1.21 | - .16 | .225 | 32 | 71 | - 0.64 | - .08 | .524 |
| Neutral - Positive | 28 | 78 | - 0.35 | - .05 | .725 | 32 | 40 | - 1.17 | - .15 | .243 |
| Do not know | | | | | | | | | | |
| Negative - Neutral | 28 | - | - | - | - | 32 | 49 | - 2.56 | - .32 | .011 |
| Negative - Positive | 28 | - | - | - | - | 32 | 40 | - 2.45 | - .31 | .014 |
| Neutral - Positive | 28 | - | - | - | - | 32 | 76 | - 0.02 | - .003 | .981 |
| Misses | | | | | | | | | | |
| Negative - Neutral | 28 | 50 | - 2.30 | - .31 | .022 | 32 | 75 | - 1.93 | - .24 | .054 |
| Negative - Positive | 28 | 71 | - 1.56 | - .21 | .119 | 32 | 59 | - 2.83 | - .35 | .005* |
| Neutral - Positive | 28 | 77 | - 1.06 | - .14 | .289 | 32 | 51 | - 2.08 | - .26 | .038 |
| | Silent | | | | | Common | | | | |
| Source incorrect | | | | | | | | | | |
| Negative - Neutral | 28 | 50 | - 0.16 | - .02 | .871 | 32 | 40 | - 0.28 | - .29 | .022 |
| Negative - Positive | 28 | 32 | - 0.96 | - .13 | .339 | 32 | 65 | - 2.02 | - .25 | .043 |
| Neutral - Positive | 28 | 31 | - 1.05 | - .14 | .292 | 32 | 26 | - 3.72 | - .47 | < .001* |
| Do not know | | | | | | | | | | |
| Negative - Neutral | 28 | - | - | - | - | 32 | 74 | - 0.50 | - .06 | .616 |
| Negative - Positive | 28 | - | - | - | - | 32 | 127 | - 0.68 | - .09 | .499 |
| Neutral - Positive | 28 | - | - | - | - | 32 | 51 | - 1.79 | - .22 | .074 |
| Misses | | | | | | | | | | |
| Negative - Neutral | 28 | 64 | - 2.07 | - .28 | .038 | 32 | 123 | - 1.61 | - .20 | .107 |
| Negative - Positive | 28 | 57 | - 2.06 | - .28 | .039 | 32 | 148 | - 0.98 | - .12 | .329 |
| Neutral - Positive | 28 | 153 | - 0.27 | - .04 | .787 | 32 | 176 | - 0.44 | - .06 | .744 |

| | Experiment 1 | | | | | Experiment 2 | | | | |
|---|---|---|---|---|---|---|---|---|---|---|
| | *n* | *T* | *z* | *r* | *p* | *n* | *T* | *z* | *r* | *p* |
| | | | | | Negative | | | | | |
| Source incorrect | | | | | | | | | | |
| Aloud-Silent/ Self - Common | 28 | 25 | - 3.18 | - .42 | .001* | 32 | 135 | - 0.11 | - .01 | .914 |
| Do not know | | | | | | | | | | |
| Aloud-Silent/ Self - Common | 28 | - | - | - | - | 32 | 88 | - 2.04 | - .26 | .042 |
| Misses | | | | | | | | | | |
| Aloud-Silent/ Self - Common | 28 | 24 | - 2.88 | - .42 | .004* | 32 | 47 | - 3.44 | - .43 | .001* |
| | | | | | Neutral | | | | | |
| Source incorrect | | | | | | | | | | |
| Aloud-Silent/ Self - Common | 28 | 56 | - 2.10 | - .28 | .036 | 32 | 64 | - 0.22 | - .03 | .830 |
| Do not know | | | | | | | | | | |
| Aloud-Silent/ Self - Common | 28 | - | - | - | - | 32 | 26 | - 3.13 | - .39 | .002* |
| Misses | | | | | | | | | | |
| Aloud-Silent/ Self - Common | 28 | 80 | - 1.77 | - .24 | .077 | 32 | 36 | - 3.28 | - .41 | .001* |
| | | | | | Positive | | | | | |
| Source incorrect | | | | | | | | | | |
| Aloud-Silent/ Self - Common | 28 | 65 | - 1.50 | - .20 | .133 | 32 | 73 | - 2.23 | - .28 | .026 |
| Do not know | | | | | | | | | | |
| Aloud-Silent/ Self - Common | 28 | - | - | - | - | 32 | 4 | - 4.00 | - .50 | <.001* |
| Misses | | | | | | | | | | |
| Aloud-Silent/ Self - Common | 28 | 56 | - 2.90 | - .39 | .004* | 32 | 41 | - 3.44 | - .43 | .001* |

*Note*. * $p < .006$

Table S7

*Output of the Model-based Bayesian Analysis ($BF_{10}$) with JASP in Experiment 1*

| Models | P(M) | P(M\|data) | $BF_M$ | $BF_{10}$ | $BF_{01}$† | error % |
|---|---|---|---|---|---|---|
| | | | Item recognition | | | |
| Null model (incl. subject) | 0.200 | 0.001 | 0.005 | 1.000 | 1.000 | |
| Source | 0.200 | 0.080 | 0.350 | 66.568 | 0.015 | 1.002 |
| Valence | 0.200 | 0.008 | 0.031 | 6.416 | 0.156 | 1.020 |
| Source + Valence | 0.200 | 0.811 | 17.196 | 671.247 | 0.001 | 2.209 |
| Source + Valence + Source ✻ Valence | 0.200 | 0.099 | 0.441 | 82.160 | 0.012 | 3.368 |
| | | | Item Br (response bias) | | | |
| Null model (incl. subject) | 0.200 | 0.016 | 0.063 | 1.000 | 1.000 | |
| Source | 0.200 | 0.145 | 0.681 | 9.314 | 0.107 | 1.189 |
| Valence | 0.200 | 0.056 | 0.236 | 3.576 | 0.280 | 0.709 |
| Source + Valence | 0.200 | 0.659 | 7.725 | 42.206 | 0.024 | 1.496 |
| Source + Valence + Source ✻ Valence | 0.200 | 0.124 | 0.568 | 7.965 | 0.126 | 2.145 |
| | | | Source recognition | | | |
| Null model (incl. subject) | 0.200 | 0.458 | 3.379 | 1.000 | 1.000 | |
| Source | 0.200 | 0.471 | 3.554 | 1.027 | 0.973 | 4.276 |
| Valence | 0.200 | 0.033 | 0.136 | 0.072 | 13.893 | 0.859 |
| Source + Valence | 0.200 | 0.034 | 0.142 | 0.075 | 13.334 | 4.479 |
| Source + Valence + Source ✻ Valence | 0.200 | 0.004 | 0.017 | 0.009 | 107.686 | 1.745 |
| | | | JOSs ratings | | | |
| Null model (incl. subject) | 0.200 | 5.501e -24 | 2.200e -23 | 1.000 | 1.000 | |
| Source | 0.200 | 0.001 | 0.005 | 2.046e +20 | 4.793e -21 | 1.211 |
| Valence | 0.200 | 2.752e -23 | 1.101e -22 | 5.003 | 0.202 | 0.962 |
| Source + Valence | 0.200 | 0.891 | 32.843 | 1.620e +23 | 5.922e -24 | 1.253 |
| Source + Valence + Source ✻ Valence | 0.200 | 0.107 | 0.482 | 1.953e +22 | 5.077e -23 | 2.279 |
| | | | Gamma correlations | | | |
| Null model (incl. subject) | 0.200 | 0.720 | 10.288 | 1.000 | 1.000 | |
| Source | 0.200 | 0.213 | 1.084 | 0.296 | 1.591e -13 | 1.183 |
| Valence | 0.200 | 0.050 | 0.209 | 0.069 | 0.088 | 0.863 |
| Source + Valence | 0.200 | 0.015 | 0.060 | 0.021 | 9.545e -16 | 1.354 |
| Source + Valence + Source ✻ Valence | 0.200 | 0.002 | 0.009 | 0.003 | 7.627e -22 | 2.856 |

*Note*. JOSs = Judgments of Source; All models include subject; † $BF_{01}$ was added to observe how the null model is being favored in relation to the remaining models.

Table S8

*Output of the Model-based Bayesian Analysis ($BF_{10}$) with JASP in Experiment 2*

| Models | P(M) | P(M\|data) | $BF_M$ | $BF_{10}$ | $BF_{01}$† | error % |
|---|---|---|---|---|---|---|
| | | | Item recognition | | | |
| Null model (incl. subject) | 0.200 | 9.313e -9 | 3.725e -8 | 1.000 | 1.000 | |
| Source | 0.200 | 0.042 | 0.174 | 4.470e +6 | 2.237e -7 | 2.179 |
| Valence | 0.200 | 5.631e -8 | 2.252e -7 | 6.046 | 0.165 | 2.280 |
| Source + Valence | 0.200 | 0.811 | 17.166 | 8.708e +7 | 1.148e -8 | 1.183 |
| Source + Valence + Source ✻ Valence | 0.200 | 0.147 | 0.691 | 1.582e +7 | 6.321e -8 | 4.872 |
| | | | Item Br (response bias) | | | |
| Null model (incl. subject) | 0.200 | 2.327e -9 | 9.309e -9 | 1.000 | 1.000 | |
| Source | 0.200 | 9.368e -4 | 0.004 | 402550.432 | 2.484e -6 | 2.121 |
| Valence | 0.200 | 3.105e -7 | 1.242e -6 | 133.435 | 0.007 | 0.952 |
| Source + Valence | 0.200 | 0.720 | 10.266 | 3.092e +8 | 3.234e -9 | 2.064 |
| Source + Valence + Source ✻ Valence | 0.200 | 0.279 | 1.551 | 1.201e +8 | 8.328e -9 | 1.817 |
| | | | Source recognition | | | |
| Null model (incl. subject) | 0.200 | 1.073e -15 | 4.292e -15 | 1.000 | 1.000 | |
| Source | 0.200 | 7.083e -5 | 2.834e -4 | 6.601e +10 | 1.515e -11 | 1.400 |
| Valence | 0.200 | 1.331e -14 | 5.323e -14 | 12.400 | 0.081 | 1.462 |
| Source + Valence | 0.200 | 0.011 | 0.044 | 1.007e +13 | 9.931e -14 | 14.643 |
| Source + Valence + Source ✻ Valence | 0.200 | 0.989 | 363.778 | 9.218e +14 | 1.085e -15 | 6.687 |
| | | | JOSs ratings | | | |
| Null model (incl. subject) | 0.200 | 2.462e -4 | 9.851e -4 | 1.000 | 1.000 | |
| Source | 0.200 | 0.304 | 1.747 | 1234.836 | 8.098e -4 | 1.029 |
| Valence | 0.200 | 2.533e -4 | 0.001 | 1.029 | 0.972 | 0.733 |
| Source + Valence | 0.200 | 0.465 | 3.477 | 1888.860 | 5.294e -4 | 1.902 |
| Source + Valence + Source ✻ Valence | 0.200 | 0.230 | 1.198 | 935.928 | 0.001 | 2.137 |
| | | | Gamma correlations | | | |
| Null model (incl. subject) | 0.200 | 0.734 | 11.031 | 1.000 | 1.000 | |
| Source | 0.200 | 0.210 | 1.066 | 0.287 | 3.489 | 0.718 |
| Valence | 0.200 | 0.042 | 0.178 | 0.058 | 17.270 | 1.063 |
| Source + Valence | 0.200 | 0.012 | 0.049 | 0.016 | 61.245 | 1.419 |
| Source + Valence + Source ✻ Valence | 0.200 | 0.001 | 0.005 | 0.002 | 571.741 | 3.106 |

*Note*. JOSs = Judgments of Source; All models include subject; † $BF_{01}$ was added to observe how the null model is being favored in relation to the remaining models.

# Method

## Data analysis

### Multinomial models.

The first step was to bring together all the participants' responses in a 3 x 4 table, where rows correspond to the source testing responses ('read in silence'; 'read aloud'; 'new') and the columns correspond to participants' responses ('read in silence'; 'read aloud'; 'read, but do not know if silently/aloud'; 'new'). This table was computed for each valence condition (see Table S2 and S3). The model adopted here followed Leshikar and colleagues (2015), which relied on the proposal of Batchelder and Riefer (1990). Accordingly, the following parameters were considered: 'D' as the probability of correctly recognizing studied stimuli irrespective of the encoding source; 'd' as the probability of correctly recognizing the stimulus source given that it was accurately identified as a studied item; 'b' as the probability of correctly guessing whether a previously studied item was studied or the probability of erroneously considering a new stimulus as old; 'g' as the probability of guessing the stimulus source given that the item was already assessed as old; 'a' as the probability of guessing the source given that the stimulus was correctly detected as study items. By imposing the constraint 'a' = 'g' (Batchelder & Riefer, 1990; Dodson, Holland, & Shimamura, 1998; Leshikar et al., 2015), eight parameters were estimated for each valence condition – two 'D', two 'd', three 'a', one 'b' – giving rise to a 24-parameter full model. Of note, two additional restrictions were imposed: all the parameter values could only vary between 0.00000001 and 0.999999999 (Dodson, Prinzmetal, & Shimamura, 1998). As we had three 'a'/'g" parameters to estimate, their sum was constrained to one. Both parameters' estimation and model fit were computed using the excel solver function following Dodson, Prinzmetal et al. (1998), which employs the maximum likelihood ratio and the likelihood statistic ($G^2$). Additionally, goodness of fit for different models was assessed by comparing $G^2$ statistics with a chi-square

distribution (alpha = .05). After an initial parameter estimation for the full 24 parameters' model, the goodness of fit of the general model was tested by changing the parameters until a satisfactory solution was found. Then, we compared the goodness of fit between different nested models in a two by two fashion, contemplating item/source memory accuracy and item/source memory response bias. As stated by Dodson, Holland et al. (1998), the idea is to compare models in which the parameters can vary without restrictions with models in which specific parameters are constrained to be equal. If the model fit does not differ significantly between models, it might be the case that the parameters are not different; if the model with free parameters reveals a better fit than the restricted one, it may suggest that the parameters are different.

**Goodman-Kruskal gamma correlation.**

To compute gamma, the following elements were considered: (a) the number of correct "remember" predictions by adding the cases that received a rating between 4 and 6; (b) the number of incorrect "remember" predictions by adding the cases with ratings between 4 and 6 that were later forgotten; (c) the number of incorrect "forget" predictions by adding the cases with ratings between 1 and 3 that were later remembered; (d) the number of correct "forget" predictions by adding the cases with ratings between 1 and 3 that were actually forgotten. These frequencies were then inserted into the following formula: $G = (ad - bc)/(ad - bc)$. However, it was not always possible to calculate the formula as some of the elements were equal to zero. To overcome this issue, an adjustment was employed as recommended by Snodgrass and Corwin (1988), and as adopted by previous studies (e.g., Bastin et al., 2012; Grainger, Williams, & Lind, 2016). More specifically, the value of 0.5 was added to each prediction frequency (a, b, c, d), and the result was then divided by the total number of judgments plus one ($N + 1$).

Gamma correlations provide a measure of association between the predictions about which words will be later remembered and forgotten and the actual performance of the participant. Large and positive gamma values are indicative of a good metamnemonic resolution, whereas values equal or below zero do not support an accurate relation between prediction and performance.

## Results

The multinomial model results and specific statistical analyses performed on the response times and confidence ratings are presented for both Experiment 1 and Experiment 2. The descriptive statistics are shown in Table S5. Additionally, for both experiments, the proportion of incorrect source responses, do not know responses, misses, correct rejections, and (corrected) false alarms were subjected to a 3 (valence: negative vs. neutral vs. positive) x 2 (source: aloud vs. silent) Friedman's ANOVA. Wilcoxon tests with Bonferroni corrections were used as follow-up tests in the case a statistically significant result was obtained in the context of the Friedman's ANOVA. Non-parametric tests were used given that most of the experimental conditions revealed a non-normal distribution according to the Shapiro-Wilk test and the absolute values of skewness and kurtosis.

Of note, a one sample *t*-test (test value = 0) was computed for each recognition measure from Experiment 1 to ensure participants could discriminate between old and new items, as well as between the two sources [$t(27) \geq 8.65$, $p < .001$ for all experimental conditions]. The same was done in the case of Experiment 2, and a similar result was observed [$t(31) \geq 5.62$, $p < .001$ for all experimental conditions].

**Experiment 1**

**Multinomial model results.** After running the solver function on the 24-parameter model, it was possible to verify that the solution was not a good fit for the data, because the obtained $G^2$ value of 25.35 was above the critical chi-square value of 12.59 (considering six degrees of freedom). To obtain a $G^2$ value below 12.59, we tried to keep most of the parameters yielded by the initial solution and changed three 'a'/'g' parameters of only one valence condition. This alteration resulted in a $G^2$ of 12.42 which is below the critical chi-square value. All the parameter values are presented in Table S4. With this model, the ANOVA results were tested.

In the case of item memory, when both positive and negative parameters were set to be equal, the $G^2$ was 20.06, which suggests that the parameters might be different after all, $G^2(2) = 7.64$, $p < .05$. When both aloud *vs.* silence conditions were equated, the $G^2$ was 38.63, which supports the ANOVA result that words read aloud are better recognized than words read silently, $G^2(3) = 26.21$, $p < .05$. In the case of source memory and considering no statistically significant differences emerged from the repeated-measures ANOVA, we set all the source 'd' to be equal regardless of the experimental condition, and the $G^2$ value obtained was 17.80. This result indicates that the parameters are not different, $G^2(5) = 5.38$, $p < .05$, in good agreement with the ANOVA outcome. Overall, the multinomial-based results are consistent with the ANOVA results.

**Incorrect source responses.** The 3 x 2 Friedman's ANOVA yielded a statistically significant result, $X^2(5) = 17.25$, $p = .004$. Specifically, the Wilcoxon tests with Bonferroni corrections ($p < .006$) showed that the proportion of incorrect responses was higher for negative words read aloud than for negative words read silently. No other comparisons reached the statistically significant threshold (see Table S6).

**Do not know responses.** The Friedman's ANOVA was not statistically significant, $X^2(5) = 5.15$, $p = .398$.

**Misses.** The Friedman's ANOVA was statistically significant, $X^2(5) = 24.11$, $p < .001$. Only two comparisons survived the Wilcoxon tests with Bonferroni corrections ($p < .006$): the proportion of misses was higher for both negative and positive words read silently during the study phase when compared to negative and positive words read aloud, respectively (see Table S6).

**Correct rejections and corrected false alarm rates.** Regarding the proportion of correct rejections, no statistically significant difference emerged, $X^2(2) = 5.93$, $p = .052$. Considering the proportion of corrected false alarm rates, the The Friedman's ANOVA showed a statistically significant effect, $X^2(2) = 6.17$, $p = .046$. Nonetheless, when applying the Wilcoxon tests with Bonferroni corrections ($p < .017$), none of the comparisons survived this correction. Taken together, no significant differences were observed between the responses to negative/neutral/positive new words during the recognition test.

**Response time.**

***JOSs ratings.*** A 3 (valence: negative, neutral, positive) x 2 (source: silent, aloud) repeated-measures ANOVA yielded no statistically significant results (valence: $F(2, 54) = 1.37$, $p = .263$, $\eta^2_p = .05$; source : $F(1, 27) = 2.90$, $p = .100$, $\eta^2_p = .10$; valence x source: $F(2, 54) = 0.12$, $p = .884$, $\eta^2_p = .01$). Thus, it seems that participants took the same time to judge if they would later recall information differing in valence and production mode (see Table S5).

***Source correct judgments.*** By applying the same 3 x 2 repeated-measures ANOVA on correct source judgments, a main effect of source, $F(1, 25) = 6.55$, $p = .017$, $\eta^2_p = .21$, and a

main effect of valence, $F(2, 50) = 4.64$, $p = .021$, $\eta^2_p = .16$, $\varepsilon = .82$, were observed. However, there was no interaction effect, $F(2, 50) = 0.10$, $p = .869$, $\eta^2_p = .004$, $\varepsilon = .80$. Specifically, the response time was slower in the case of positive words (*M* = 3722, *SE* = 256) when compared with neutral words (*M* = 3231, *SE* = 152, *p* = .022). The response time was also slower for words read silently (*M* = 3641, *SE* = 186) than words read aloud (*M* = 3257, *SE* = 189, *p* = .017).

***Correct rejections.*** In the case of the response time for correct rejections of new words, a repeated measures ANOVA with the factor valence showed a statistically significant effect, $F(2, 54) = 4.80$, $p = .012$, $\eta^2_p = .16$, revealing once again that the time to respond to new positive words (*M* = 2319, *SE* = 115) was slower in comparison with new neutral words (*M* = 2039, *SE* = 89, *p* = .015).

**Confidence ratings.**

***Source correct judgments.*** When analyzing the confidence ratings after accurate source memory judgments, the 3 x 2 repeated-measures ANOVA revealed only a statistically significant main effect of source, $F(1, 26) = 6.99$, $p = .014$, $\eta^2_p = .21$, (valence: $F(2, 54) = 1.37$, $p = .263$, $\eta^2_p = .05$; valence x source: $F(2, 54) = 0.12$, $p = .884$, $\eta^2_p = .01$): confidence ratings for words read aloud (*M* = 5.34, *SE* = 0.08) were higher than confidence ratings for words read silently (*M* = 5.07, *SE* = 0.12, *p* = 0.14).

***Correct rejections.*** In the case of confidence ratings for accurately rejected new words, a repeated-measures ANOVA run on the factor valence yielded a significant effect, $F(2, 54) = 12.57$, $p < .001$, $\eta^2_p = .32$, $\varepsilon = .81$, which showed that participants were more confident when rejecting both new negative (*M* = 5.07, *SE* = 0.14, *p* = .001) and new neutral words (*M* = 5.03, *SE* = 0.14, *p* = .004) than new positive words (*M* = 4.75, *SE* = 0.16, *p* = 0.14).

**Experiment 2**

**Mean proportion of "yes" responses.** The mean proportion of "yes" responses in the self-referential condition was .09 (*SD* = .09), .31 (*SD* = .18), and .69 (*SD* = .17) for negative, neutral, and positive words, respectively. In the case of the common condition, this proportion was .64 (*SD* = .16), .78 (.16), .90 (.12) for negative, neutral, and positive words, respectively. A 3 (valence: negative vs. neutral vs. positive) x 2 (source: self-reference vs. common) repeated-measures ANOVA revealed a main effect of valence, $F(2, 62) = 231.46$, $p < .001$, $\eta^2_p = .88$, a main effect of source, $F(1, 31) = 263.91$, $p < .001$, $\eta^2_p = .90$, and an interaction effect, $F(2, 62) = 20.84$, $p < .001$, $\eta^2_p = .40$. In general, the mean proportion of "yes" responses was higher in the case of common judgments compared to self-referential judgments (all $p < .001$), and it also differed according to word valence: negative < neutral < positive (all $p < .01$).

**Multinomial model results.** When modelling the results of this experiment based on the 24-parameter model, we came across the same problem reported in Experiment 1, that is, the solution found with solver was not the best fit for the data as the $G^2$ value of 44.39 was above the critical chi-square value of 12.59 (considering six degrees of freedom). In these circumstances, we applied the same strategy reported in Experiment 1 to achieve a better solution. We ended up obtaining a $G^2$ of 11.15 which is below the critical chi-square value. All the parameter values are presented in Table S4. In the case of item memory, we started by testing if there was a difference between the parameters of neutral and negative stimuli. For this, negative and neutral parameters were set to be equal regardless of the source condition. The obtained $G^2$ value was 43.46 which supports the difference between neutral and negative words, $G^2(3) = 32.31$, $p < .05$. To confirm the main effect of source (self-reference vs. common), the item parameters were set to be equal for each valence condition, which

revealed a $G^2$ value of 71.23. So, the model that posits the source conditions as different is a better fit for the data, $G^2(3) = 60.08$, $p < .05$, which is in accordance with the ANOVA results. Concerning the source memory accuracy results, specifically in the context of the self-referential condition, the 'd' parameter was set to be equal for both neutral and positive stimuli. The $G^2$ value was 11.85, which suggests that these parameters might not differ, $G^2(1) = 0.70$, $p < .05$. Once again, this result supports the ANOVA results showing that the source of both neutral and positive stimuli is better recognized than the source of negative stimuli. In the case of the common condition, the ANOVA results showed that the source of emotional words was less accurately recognized when compared with neutral stimuli. Thus, we tested a model wherein both negative and positive 'd' parameters of the common condition were equal while keeping the 'd' neutral parameter varying freely. The obtained $G^2$ value was 14.02, which shows that the former parameters do not differ, $G^2(1) = 2.87$, $p < .05$. Again, the multinomial-based results are consistent with the ANOVA results.

**Incorrect source responses.** The 3 x 2 Friedman's ANOVA revealed a statistically significant effect, $X^2(5) = 26.80$, $p < .001$. Nonetheless, only one comparison survived the statistical significance threshold imposed by the Wilcoxon tests with Bonferroni corrections ($p < .006$). Specifically, the proportion of incorrect source responses for positive words encoded in the common condition was higher in comparison with neutral words in the common condition (see Table S6).

**Do not know responses.** The 3 x 2 Friedman's ANOVA was statistically significant, $X^2(5) = 36.98$, $p < .001$. The Wilcoxon tests with Bonferroni corrections ($p < .006$) showed that for both neutral and positive words studied in the common condition received more 'do

not know’ responses in contrast to neutral and positive words in the self-referential condition, respectively (see Table S6).

**Misses.** A statistically significant result was obtained with the Friedman’s ANOVA, $X^2(5) = 45.08$, $p < .001$. In the case of words studied self-referentially, the proportion of misses was higher in the case of negative words in comparison with positive words. Moreover, irrespective of valence, words encoded in the common condition presented higher proportion of misses in contrast to words studied in the self-referential condition (see Table S6).

**Correct rejections and corrected false alarm rates.** In the case of correct rejections, the Friedman’s ANOVA yielded a statistically significant result, $X^2(2) = 9.60$, $p = .008$. During the test phase, participants correctly identified more new neutral words as ‘new’ when compared to emotionally laden words (negative vs. neutral: $T = 44$, $z = - 2.89$, $p = .004$, r = - .36; positive vs. neutral: T = 57, $z = - 3.05$, $p = .002$, r = - .38). Concerning the false alarms, a similar result was obtained, $X^2(2) = 9.19$, $p = .010$., as new emotional words demonstrated higher false alarm rates than new neutral words (negative vs. neutral: $T = 54$, $z = - 2.87$, $p = .004$, $r = - .36$; positive vs. neutral: T = 58, $z = - 3.04$, $p = .002$, $r = - .38$).

**Response time.**

***JOSs ratings.*** A 3 (valence: negative, neutral, positive) x 2 (source: silent, aloud) repeated-measures ANOVA revealed only a main effect of valence, $F(2, 62) = 5.74$, $p = .009$, $\eta^2_p = .16$, $\varepsilon = .83$; the main effect of source, $F(1, 31) = 0.24$, $p = .627$, $\eta^2_p = .01$, and the interaction between both factors, $F(2, 62) = 0.85$, $p = .431$, $\eta^2_p = .03$, were not statistically significant. This effect revealed that participants were faster in the metamemory evaluation of

positive stimuli (*M* = 1630, *SE* = 102) when compared to both negative (*M* = 1829, *SE* = 115, *p* = .007) and neutral stimuli (*M* = 1857, *SE* = 138, *p* = .043).

***Source correct judgments.*** The 3 x 2 repeated-measures ANOVA showed a main effect of source, $F(1, 31) = 30.12$, $p < .001$, $\eta^2_p = .49$, and a main effect of valence, $F(2, 62) = 4.23$, $p = .019$, $\eta^2_p = .12$, but no interaction effect, $F(2, 62) = 1.03$, $p = .362$, $\eta^2_p = .03$. Accurate source judgments were faster in the context of neutral (*M* = 3149, *SE* = 156) compared to negative words (*M* = 3422, *SE* = 197, *p* = .016). Furthermore, accurate source responses were also faster for words encoded in the self-referential condition (*M* = 2987, *SE* = 154) when compared to words encoded in the common condition (*M* = 3520, *SE* = 178, $p < .001$).

***Correct rejections.*** Considering the response time for correct rejections, the repeated-measures ANOVA yielded a statistically significant effect of valence, $F(2, 62) = 4.84$, $p = .011$, $\eta^2_p = .14$: participants were faster in the rejection of neutral (*M* = 2131, *SE* = 94) compared to positive words (*M* = 2373, *SE* = 117, *p* = .037).

**Confidence ratings.**

***Source correct judgments.*** The analysis of the confidence ratings of accurately identified sources showed a main effect of source, $F(1, 31) = 16.32$, $p < .001$, $\eta^2_p = .35$, and a main effect of valence, $F(2, 62) = 6.22$, $p = .003$, $\eta^2_p = .18$, but no statistically significant interaction effect, $F(2, 62) = 1.17$, $p = .317$, $\eta^2_p = .04$. The confidence ratings of both emotional words (negative: *M* = 5.10, *SE* = 0.14, *p* = .034; positive: *M* = 5.10, *SE* = 0.15) was significantly lower than the confidence ratings of neutral words (*M* = 5.29, *SE* = 0.14, *p* = .011).

***Correct rejections.*** In the case of confidence ratings after correct rejections, a main effect of valence was observed, $F(2, 62) = 19.78$, $p < .001$, $\eta^2_p = .39$, showing statistically

significant differences between all valence conditions ($p$ < .01): neutral ($M$ = 4.96, $SE$ = 0.14) > negative ($M$ = 4.80, $SE$ = 0.15) > positive ($M$ = 4.63, $SE$ = 0.16).

References


Bastin, C., Feyers, D., Souchay, C., Guillaume, B., Pepin, J. L., Lemaire, C., … Salmon, E. (2012). Frontal and posterior cingulate metabolic impairment in the behavioral variant of frontotemporal dementia with impaired autonoetic consciousness. *Human Brain Mapping, 33*(6), 1268–1278. http://doi.org/10.1002/hbm.21282

Batchelder, W. H., & Riefer, D. M. (1990). Multinomial processing models of source monitoring. *Psychological Review, 97*(4), 548-564. http://doi.org/10.1037/0033-295X.97.4.548

Dodson, C. S., Holland, P. W., & Shimamura, A. P. (1998). On the recollection of specific- and partial-source information. *Journal of Experimental Psychology: Learning, Memory, and Cognition, 24*(5), 1121-1136. http://doi.org/10.1037//0278-7393.24.5.1121

Dodson, C. S., Prinzmetal, W., & Shimamura, A. P. (1998). Using excel to estimate parameters from observed data: An example from source memory data. *Behavior Research Methods, Instruments, & Computers, 30*(3), 517-526.

Grainger, C., Williams, D. M., & Lind, S. E. (2016). Judgment of learning accuracy in high-functioning adolescents and adults with autism spectrum disorder. *Journal of Autism and Developmental Disorders*, *46*(11), 3570–3582. http://doi.org/10.1007/s10803-016-2895-1

JASP Team (2018). JASP (Version 0.9.0.1) [Computer software].

Leshikar, E. D., Dulas, M. R., & Duarte, A. (2015). Self-referencing enhances recollection in both young and older adults. *Neuropsychology, Development, and Cognition. Section*

*B, Aging, Neuropsychology and Cognition*, *22*(4), 388–412. http://doi.org/10.1080/13825585.2014.957150

Snodgrass, J. G., & Corwin, J. (1988). Pragmatics of measuring recognition memory: Applications to dementia and amnesia. *Journal of Experimental Psychology: General*, *117*(1), 34–50. http://doi.org/10.1037/0096-3445.117.1.34
